# Why Are Medical and Health-Related Studies Not Being Published? A Systematic Review of Reasons Given by Investigators

**DOI:** 10.1371/journal.pone.0110418

**Published:** 2014-10-15

**Authors:** Fujian Song, Yoon Loke, Lee Hooper

**Affiliations:** Department of Population Health & Primary Care, Norwich Medical School, Faculty of Medicine and Health Science, University of East Anglia, Norwich, Norfolk, United Kingdom; Tilburg University, Netherlands

## Abstract

**Objective:**

About half of medical and health-related studies are not published. We conducted a systematic review of reports on reasons given by investigators for not publishing their studies in peer-reviewed journals.

**Methods:**

MEDLINE, EMBASE, PsycINFO, and SCOPUS (until 13/09/2013), and references of identified articles were searched to identify reports of surveys that provided data on reasons given by investigators for not publishing studies. The proportion of non-submission and reasons for non-publication was calculated using the number of unpublished studies as the denominator. Because of heterogeneity across studies, quantitative pooling was not conducted. Exploratory subgroup analyses were conducted.

**Results:**

We included 54 survey reports. Data from 38 included reports were available to estimate proportions of at least one reason given for not publishing studies. The proportion of non-submission among unpublished studies ranged from 55% to 100%, with a median of 85%. The reasons given by investigators for not publishing their studies included: lack of time or low priority (median 33%), studies being incomplete (median 15%), study not for publication (median 14%), manuscript in preparation or under review (median 12%), unimportant or negative result (median 12%), poor study quality or design (median 11%), fear of rejection (median 12%), rejection by journals (median 6%), author or co-author problems (median 10%), and sponsor or funder problems (median 9%). In general, the frequency of reasons given for non-publication was not associated with the source of unpublished studies, study design, or time when a survey was conducted.

**Conclusions:**

Non-submission of studies for publication remains the main cause of non-publication of studies. Measures to reduce non-publication of studies and alternative models of research dissemination need to be developed to address the main reasons given by investigators for not publishing their studies, such as lack of time or low priority and fear of being rejected by journals.

## Introduction

About half of medical and health related research studies remain not published [1], [2], and there is still ongoing debate about whether publishing selectively could be justifiable [3], [4]. However, research ethical obligations require the appropriate dissemination and publication of all research results [5]. When research methods and results are inaccessible to users of research, the investment in such research contributes little or nothing to knowledge or practice. Failure to publish research can potentially be regarded as a form of research misconduct and unethical [6]. In addition, empirical evidence indicates the existence of research dissemination bias, as published studies tend to be systematically different from unpublished studies [1], [7]. Adverse consequences of research inaccessibility include unnecessary duplication, harm to patients, waste of limited resources, and loss of (trust in) scientific integrity [1], [8].

The dissemination profile of research is influenced by the interests of a variety of different stakeholders [8]. For example, there have been a number of high profile cases in which unfavourable results of studies sponsored by pharmaceutical companies were publically inaccessible for commercial reasons [1], [9]. Rejection by journals may also be a cause of non-publication of studies. However, evidence indicates that many studies remain unpublished because researchers failed to write up and submit their work to journals for publication [10]–[12]. Researchers are motivated to publish as many studies as possible because of a “publish or perish” culture [13], [14]. It is therefore surprising that investigators who need to publish are the main cause of non-publication of studies.

There are many recently published survey studies that have reported reasons given by researchers describing why they failed to publish their work in peer-reviewed journals. We conducted a systematic review of relevant surveys, in order to improve our understanding of reasons given by investigators for not publishing studies, and help to develop innovative, new or better measures to reduce non-publication of completed research.

## Methods

We searched MEDLINE, EMBASE, PsycINFO, and SCOPUS for relevant reports until 13^th^ September 2013 (see Appendix S1 for the search strategy). References of retrieved articles and reviews on publication bias were also examined for relevant studies. Titles and abstracts of retrieved citations from the searches of electronic databases were screened by two independent reviewers. Full text articles of possibly relevant studies were assessed by one reviewer to identify eligible studies.

We included any reports of surveys that provided data on reasons given by investigators for not publishing studies they conducted. Articles that discussed reasons for not publishing studies in general but did not provide empirical data on reasons given by investigators were excluded. There were no restrictions on languages or publication status. We used Google Translate to obtain key information from studies published in languages other than English.

Data extraction was conducted by one reviewer (FS), and checked by a second reviewer (YL or LH). From the included survey reports, we extracted the following data: source and types of unpublished studies (conference abstracts, study protocols, survey of academics or professionals, postgraduate submissions, rejected manuscripts, study sponsors, and trial registries), survey methods, response rate, number of unpublished studies, number of unsubmitted studies, stated reasons for not publishing.

Methods used to categorise reasons for not publishing may be different across included surveys. Stated reasons for not publishing were grouped into categories: including lack of time or low priority, unimportant or negative results, journal rejection, fear of being rejected, and so on. We used the number of unpublished studies as the denominator to calculate the proportion of non-submission and specific reasons for non-publication for each included survey reports. First, proportions of reasons reported in survey reports were transformed to normally distributed values using the Freeman-Tukey transformation methods [15], [16]. The normally distributed transformed proportions were then used to estimate 95% confidence intervals and for meta-regression analyses. The use of transformed proportions for estimating confidence intervals also avoided the possibility of inappropriate results where the lower limit is below zero or the upper limit exceeds one, when the point proportion is approaching 0% or 100%.

Because of significant heterogeneity across reports, quantitative pooling of results was not conducted. We used forest plots to visually present individual results of included reports, and presented medians and ranges (minimum to maximum) of reported proportions of reasons given for non-publication.

For exploratory subgroup analysis, included reports were separated into subgroups by types of unpublished studies (abstracts vs. protocols or other), design of unpublished studies (clinical trials only vs. other or different designs), period of surveys conducted (before 2000, from 2000 to 2004, and since 2005), rate of non-submission, and response rate. The differences between subgroups were statistically tested using univariate, random-effects meta-regression (Stata/IC 12.1 for Windows “metareg” command), and the Bonferroni correction was used for multiple statistical testing.

## Results

**Figure 1:**
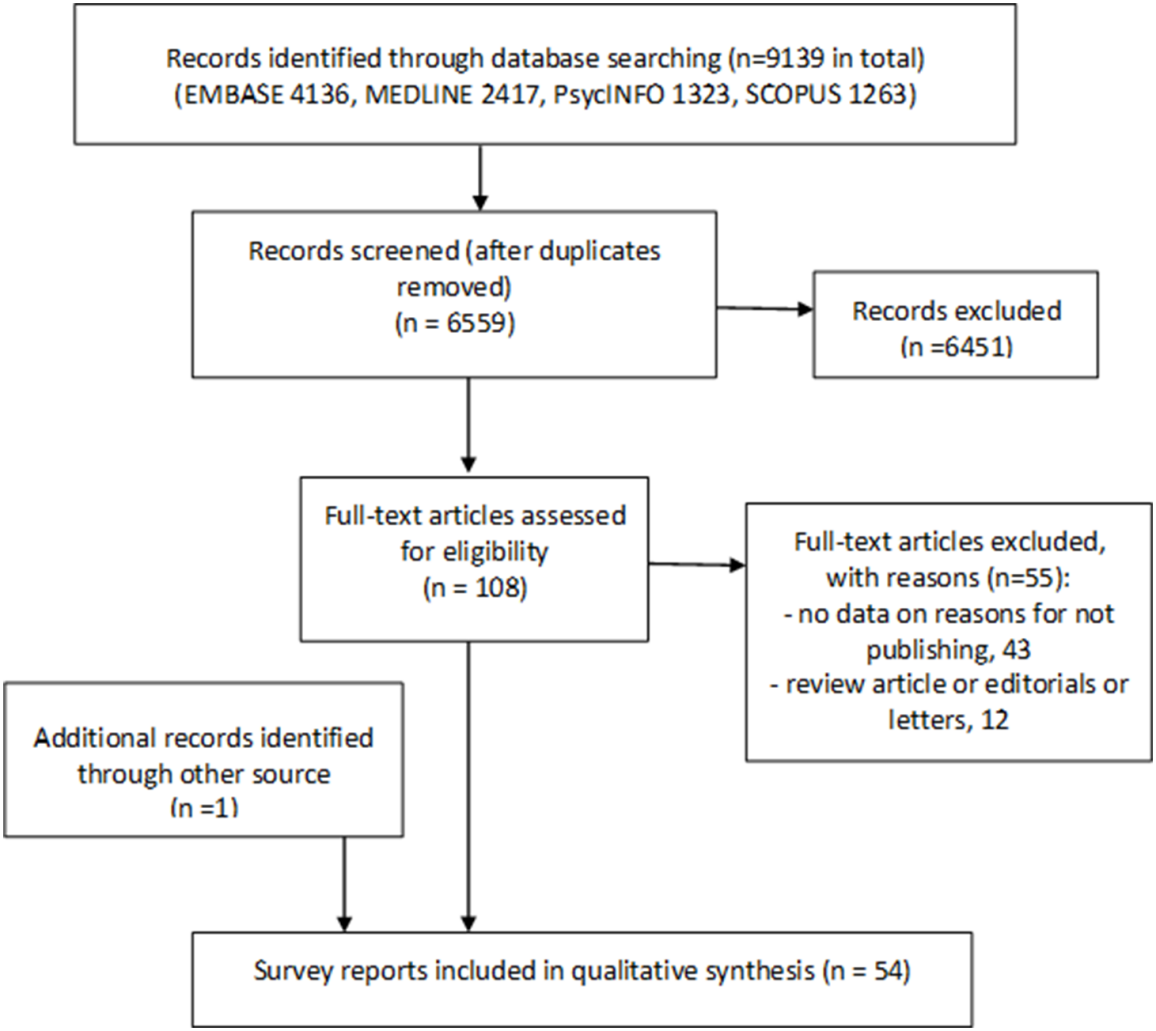
Flow diagram for study selections.

The process of study selection is shown in Figure 1. By checking titles and abstracts of retrieved citations (n = 6559), we identified 108 citations that were possibly relevant. After examining the 108 full-text articles, we included 53 reports that provided investigator’s reasons for not publishing their studies [10], [11], [17]–[67]. One additional report was identified by an informal search of PubMed [68], because the formal search excluded titles that used terms “meta-analysis” or “systematic review” (See Appendix S1). We included 54 survey reports in total.

The main characteristics of the included reports are presented in Appendix S2. Non-publication was generally defined as lack of full publication in peer-reviewed journals. Of the 54 included reports, 10 surveys were conducted before 2000, 17 conducted from 2000 to 2004, and 27 conducted since 2005. There were 27 surveys of authors of unpublished conference abstracts, 11 reports in which unpublished studies were revealed by academics or professionals who responded to a survey, 7 reports in which unpublished studies were identified from protocol cohorts, 4 surveys of postgraduate submissions, two surveys of studies sponsored by a funding body, two surveys of rejected manuscripts, and one survey of studies from a trial registry.

There were 38 reports that included unpublished studies with a mix of different designs or types, 11 reports that included only unpublished trials, and one each for unpublished animal research, epidemiological research, qualitative research, methodological research, and systematic reviews (Appendix S2).

Survey methods included mainly postal or email questionnaires, and telephone or face-to-face interviewing. The response rate by authors ranged from 8% to 100%, with a median of 64% (Appendix S2). According to 43 reports with sufficient data, the median number of unpublished studies was 65 (ranged from 7 to 223). Of the 54 included reports, 38 provided sufficient data to estimate proportions of at least one stated reason.

### Main reasons for non-publication

Table 1 presents the medians and ranges of reported proportions of reasons for non-publication. Estimated proportions (with 95% confidence intervals) of reasons for non-publication from individual reports and results of statistical tests for heterogeneity are shown in Appendix S3 (forest plots).

**Table 1:** Reasons given by investigators for non-publication of studies –summary of results of included surveys.

Reasons	No. ofsurveys thatreportedthe reason	Total no. of statedreason/unpublishedstudies	Reported proportion:median (range)
Non-submission	30	2156/2592	85% (55%, 100%)
Study incomplete or ongoing	18	273/1509	15% (3%, 56%)
In preparation or under review	22	293/1778	12% (3%, 65%)
Study not for publication	9	107/880	14% (3%, 38%)
Similar findings published	10	52/867	5% (3%, 13%)
Submission rejected by journal	25	210/2197	6% (2%, 27%)
Fear of being rejected	9	110/926	12% (6%, 26%)
Lack of time or low priority	32	873/2634	33% (11%, 60%)
Results not important or negative	19	293/1593	12% (1%, 34%)
Poor study quality or design	16	197/1611	11% (2%, 32%)
Sponsor/funder problems	4	31/230	9% (5%, 24%)
Author/co-author problems	14	156/1337	10% (4%, 23%)

**Figure 2:**
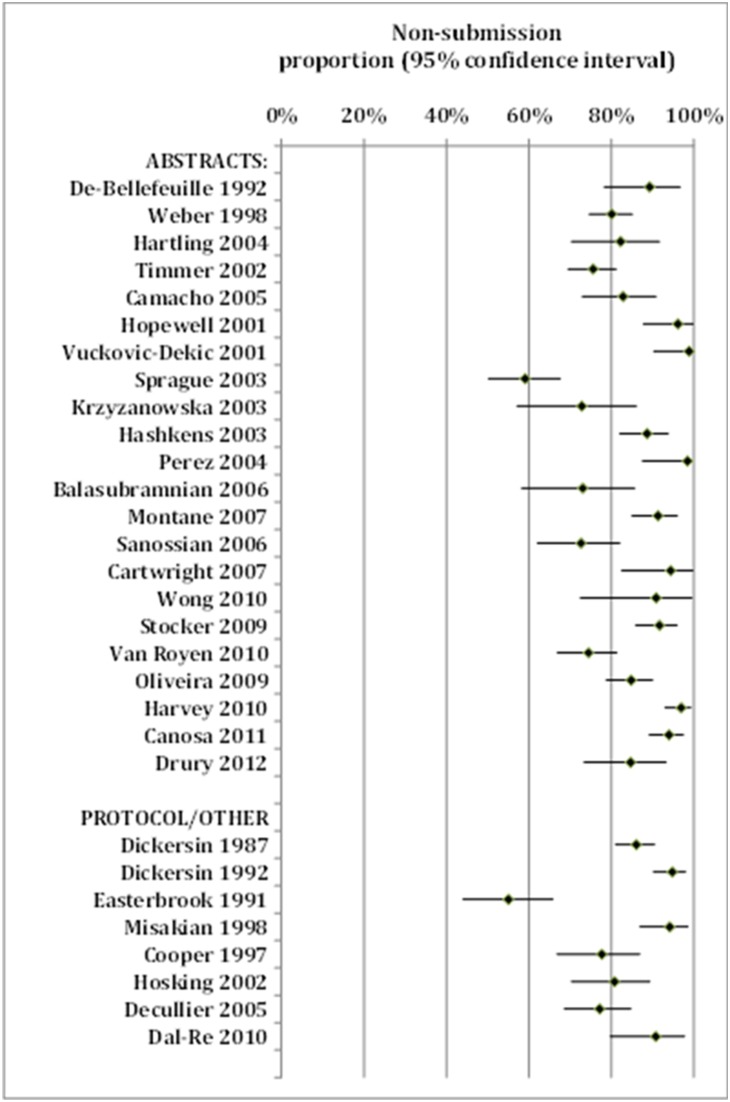
Proportions of non-submission among unpublished studies – results of individual surveys.

Two reports included only studies that had been rejected for publication by journals [35], [46]. Using data from the other 30 reports, the proportion of non-submission among unpublished studies ranged from 55% to 100%, with a median of 85% (Table 1 and Figure 2).

The most commonly stated reason for non-publication was lack of time or low priority (median 33%, range: 11% to 60%) (Table 1). Other important reasons for non-publication included: studies being incomplete or still ongoing (median 15%), study not for publication (median 14%), manuscript in preparation or under review (median 12%), unimportant or negative result (median 12%), poor study quality or design (median 11%), fear of rejection (median 12%), rejection by journals (median 6%), author or co-author problem (median 10%), and sponsor or funder problem (median 9%).

Findings from the included reports in which data were insufficient for quantitative analyses were qualitatively consistent with the above results. For example, lack of time was often mentioned as an important reason for non-publication of studies (Appendix S2).

### Heterogeneity in reasons given for non-publication

There was substantial heterogeneity in the proportion of reasons given for non-publication across individual reports (Table 1 and Appendix S3). The results of exploratory subgroup analyses are presented in Appendix S4. In most cases, heterogeneity across reports could not be explained by sources of unpublished studies, study design, period of surveys, response rate, or non-submission rate (Appendix S4). There were four statistically significant findings (*P*<0.05) out of a total of 57 statistical tests of subgroup differences. If a Bonferroni correction was used for multiple statistical testing, only one of the subgroup analyses retained statistical significance. A higher proportion of non-submission was statistically significantly associated with a lower proportion of journal rejection (after the Bonferroni correction, *P*<0.01) as a reason for non-publication, which is expected as unsubmitted manuscripts cannot be rejected by journals. Specifically, journal rejection as a reason for non-publication was reduced by 0.7%, with a 10% increase in non-submission. For the three other subgroup analyses with *P*<0.05, subgroup differences became statistically non-significant with the Bonferroni correction for multiple testing (see Appendix S4 for details).

## Discussion

Our systematic review is the most comprehensive review of reasons given by investigators for not publishing studies. As 85% of unpublished studies have not been submitted to journals, non-publication of many studies was directly caused by failure of authors to write up and submit their work to journals. The most commonly stated reason for non-publication was lack of time or low priority. Other stated reasons included unimportant or negative result, study not completed, study not for publication, fear of journal rejection, poor study quality or design, manuscript in preparation or under review, author or co-author problem, sponsor or funder problem.

### Acceptability of reasons given for not publishing studies

Research dissemination will be a biased process when non-publication is due to negative results, which is clearly unacceptable [8]. Some of the stated reasons seem acceptable for non-publication of a study, such as manuscript in preparation or under review, or the study being still ongoing. However, the acceptability of most stated reasons is disputable in terms of potential bias in the research literature and the ethics of scientific conduct [69], [70].

Except for two reports that included only studies rejected by a journal, journal rejection was an infrequently stated reason for non-publication (median 6%). Rejection by a journal may not necessarily be an acceptable reason for non-publication, as many studies which were eventually published had previously been rejected by one or more journals. Okike et al. found that, in five years, 76% of the manuscripts rejected by the Journal of Bone and Joint Surgery (American Volume) had been published in other journals [46].

Fear of journal rejection (median 12%) was a more frequently stated reason than the actual rejection by journals (median 6%). Therefore, the low proportion of journal rejection as a reason for non-publication may be partly due to the selective submission of studies by investigators. To some extent, experienced researchers may be able to guess whether and where a study is likely to be accepted for publication. Fear of being rejected may originate from perception that results are not important or statistically non-significant, awareness of poor study quality, and similar findings already being published by others. Brice and Chalmers found that only 12% of high quality medical journals explicitly encourage authors to submit manuscripts of robust research, “regardless of the direction or strength of the results” [71]. Unfortunately, there was no direct empirical evidence to reveal why authors felt their work was unlikely to be accepted by a journal.

Lack of time or low priority (median 33%) is an arguable reason for not publishing studies. Individual researchers may become swamped if engaged in several streams of research that could potentially be developed for journal publication. It is very time-consuming to prepare, submit, revise, and re-submit manuscripts for publication. Repeated rejection by multiple journals is not unusual before a study is eventually accepted. For career advancement, rewards from publishing in high-impact journals would be greater than in low-impact journals. Many researchers may indeed have insufficient time to publish all their work in peer-reviewed journals. It is therefore practically logical for investigators to focus on “wonderful results” rather than “negative results”, as the former may be more likely to be published in a high-impact journal [20]. Fanelli found that the frequency of reporting positive results was associated with ‘the more competitive and productive academic environments’ in the United States [72].

It has been stated that some unpublished studies were not intended for publishing in peer-reviewed journals (median 14%). Postgraduate theses or dissertations are used to obtain academic degrees or professional certificates. However, there is no good reason not to publish degree theses [73]. Feasibility or pilot studies aim to help investigators to develop full scale studies, and the methods and results of such studies should be published or publically accessible to other researchers. Findings from industry sponsored trials are used to gain regulatory approvals of commercial products, and such studies should be published or publically accessible [74].

Poor study quality or design problems as a reason for non-publication (median 11%) is also arguable. It has been suggested that quality rather than the number of publications should be emphasized to measure researchers’ scientific productivity [75]. However, we need to distinguish the number of studies published and the number of studies conducted. A reduction in emphasis on the volume of published material should not be used to justify non-publication of studies that have been conducted [1], [76]. The results of poor quality studies may have no immediate impact on clinical practice and health policy. However, methodological issues or problems experienced in failed studies may inform other investigators to avoid similar mistakes or problems [77]. It may be difficult to publish failed studies in peer-reviewed journals, and alternative dissemination approaches may be required. For example, clinical trial registries may provide a conventional platform to record the methods and results of failed trials [78].

Suppression of unfavourable results by study sponsors or funders has always been a concern [8]. However, only four surveys provided data on the proportion of sponsor or funder problem as a reason for non-publication [21], [34], [39], [68]. A survey of study protocols with different designs, conducted in 1990, found that 24% of the 78 unpublished studies were due to sponsor control of data [34]. The three more recent studies reported lower proportions of sponsor or funder problem as a reason for non-publication (5%, 7%, and 11% respectively) [21], [39], [68]. Non-publication of studies because of sponsors or funders was considered to be rare but important in another three studies that did not provide quantitative data [33], [50], [59].

Some studies were not published because other investigators had already published similar findings (median 5%). However, unnecessary duplication should be distinguished from appropriate replication. Empirical evidence does reveal the existence of unnecessary duplication in health research, and new research should be justified according to what is already known from systematically reviewing existing evidence [79]. However, with regards to completed studies, results from all primary research should be properly maintained and made publicly accessible.

Author or co-author problems were further reasons given for not publishing studies (median 9%), including job/post change, trouble with co-authors, and other’s responsibility to write up. It is unclear why a higher proportion of author problems was associated with a lower response rate, and why it was a more frequent reason for non-publication of clinical trials compared to non-publication of studies with mixed or other designs (Appendix S4). It is likely that, to some extent, author problems are associated with other stated reasons such as lack of time or low priority, unimportant results, and poor study quality. For example, if the principal investigator decides not to publish, it may be very difficult for junior co-investigators to publish results, even if they would like to [80].

### Implications for strategies to reduce non-publication of studies

Prospective registration of clinical trials at their inception has been developed in order to reduce selective publication of trials [8], [81]. However, it is currently mandatory only for certain categories of clinical trials (for example, trials of medicinal products or medical device). It is still difficult, if not impossible, to uncover unpublished observational research and basic biomedical studies. In addition to prospective registration of studies and other enforcement measures, additional measures are required, relating to the main reasons given by investigators for not publishing their studies.

Existing recommendations for reducing non-publication of studies may have failed to give sufficient consideration to commonly stated reasons, such as lack of time or low priority, by investigators for not publishing studies. For instance, the problem of lack of time needs to be addressed from the beginning to the end of a research. First, as Altman recommended, we need “less research, better research, and research done for the right reasons” [82]. Any new research should be relevant and of sufficiently high quality [79]. If national registries of funded projects (across funding bodies) were made available, research funders would be able to take into account the recent and current workload of investigators, so that they won’t simultaneously conduct too many studies. Moreover, researchers who failed to publish findings from their completed studies could be given lower priority for further funding, preferably across funding bodies. The publication rate of funded studies from the Health Technology Assessment (HTA) Programme in the UK was 98% after 2002, by publishing the results in the programme’s own journal (HTA monograph) and withholding 10% of funds until the publication of the full report [83].

Second, the process of peer-reviewed publications should be more streamlined, and modified to save investigators’, peer-reviewers, and editors’ time [84]. It would save time if the same general guidelines for manuscript submission could be adopted by different journals. Many studies are rejected by multiple journals before they are eventually accepted for publication. Effort and time required to publish studies that are likely to be rejected by multiple journals will be particularly great, because the format of a manuscript needs to be changed before submitting it to each different journal, and repeated editorial and peer-review processes by different journals are required for a single manuscript.

For some studies, publication in a conventional journal may be too time-consuming to pursue, or the possibility of being accepted by a journal is extremely slim for various reasons. Therefore, there is a need for alternative modalities to retain “unpublishable” studies so that other researchers could easily identify these studies and access their methods and results. Investigators should need much less time to publish their work by alternative approaches than in conventional journals. The publication of studies through the alternative system should be acceptable as the fulfilment of mandatory research dissemination required by research sponsors, funders, and other regulatory bodies. Given advanced information technology, the development of publication processes alternative to conventional journals is technically possible. For example, established trial registration system can be alternative models for research dissemination, whereby results can be posted together with previously registered protocols [84]. Other models for scientific communication have also been tested in some research fields [85], [86].

### Limitations of the review

Biased selection of studies for publication may be a common problem in any research fields. However, it is unclear whether the results of this systematic review are directly relevant to the non-publication of research in fields other than medical and health-related studies. Reasons for non-publication of studies may even be different within medical and health-research. Further research is required to examine the similarity and differences in research dissemination of different fields.

The quality of the included surveys was not formally assessed, as we are not aware of any validated tools to assess quality of such studies. One readily available indicator of survey quality may be the response rate by authors, which ranged from 8% to 100% (median 64%). Publication bias and outcome reporting bias is likely in the included studies. The estimated frequency of stated reasons may be exaggerated when multiple reasons reported by a small number of respondents in some surveys were lumped together into a single “other” category, which could not be used for calculating proportions. Multiple reasons for non-publication of individual studies were usually allowed in included surveys, so it is possible that the frequency of some stated reasons may have been under-estimated when investigators partially selected reasons that were interrelated. In addition, we were unable to distinguish between one-off rejections as compared to repeated rejections by journals.

This systematic review included very diverse studies in terms of sources of unpublished studies, types of unpublished studies, research fields, survey methods, selection of survey participants, and questions asked about reasons for not publishing studies. Although the generalizability of findings from this systematic review may be improved by including diverse reports, there was significant heterogeneity in results across the included reports. In general, the frequency of main reasons given for non-publication was not associated with the source of unpublished studies, study design, response rate, or time when a survey was conducted. Three of the four significant subgroup differences (at the level of *P*<0.05), from a large number of subgroup analyses, were no longer statistically significant if the Bonferroni correction was carried out. In most cases, the differences between subgroups were small and with unclear practical importance. In addition, the power of meta-regression analyses was limited due to the relatively small number of primary studies included. Therefore, results of our subgroup analyses should be interpreted with great caution [87].

### Conclusions

Non-submission of studies for publication remains the main cause of non-publication of medical and health-related studies. Measures to reduce non-publication of studies and alternative models of research dissemination need to be developed taking into account the common reasons given by investigators for not publishing their studies, such as lack of time or low priority and fear of being rejected by journals.

## Supporting Information

Appendix S1 **Literature search strategy (Ovid –MEDLINE, EMBASE).**
(PDF)

Appendix S2 **The main characteristics of reports that provided data on reasons for non-publication of studies.**
(PDF)

Appendix S3 **Forest plots of stated reasons for non-publication of studies.**
(PDF)

Appendix S4 **Results of meta-regression analyses of differences between subgroups.**
(PDF)

Checklist S1 **PRISMA Checklist.**
(PDF)
