# Supplementary material for: Why Are Medical and Health-Related Studies Not Being Published? A Systematic Review of Reasons Given by Investigators
Source: PLoS One. 2014 Oct 15;9(10):e110418. doi: 10.1371/journal.pone.0110418 (PMC4198242; doi:10.1371/journal.pone.0110418)
Supplement: Appendix S1 — Literature search strategy (Ovid –MEDLINE, EMBASE). (PDF) [file pone.0110418.s001.pdf]

Appendix S1: Literature search strategy (Ovid –MEDLINE, EMBASE)

1. publication bias/
2. (file adj1 drawer*).ti,ab.
3. ((rate* or full or fully or journal* or peer-reviewed) adj3 (publish* or publication* or non-publication* or nonpublication* or unpublish* or unpublish*)).ti,ab.
4. ((fate or publish* or unpublish* or un-publish* or publication* or nonpublication* or non-publication*) adj4 (abstracts or presentation* or meeting* or conference* or congress*)).ti,ab.
5. 1 or 2 or 3 or 4
6. ((survey* or interview* or questionnaire*) and (author* or researcher* or investigator* or trialist*)).ti,ab.
7. 5 and 6
8. ((factor* or determinant* or reason*) adj4 (publish* or publication* or nonpublication* or non-publication* or unpublish* or unpublish*)).ti,ab.
9. 7 or 8
10. (meta-analysis or systematic review).ti.
11. 9 not 10
