# Supplementary material for: Why Are Medical and Health-Related Studies Not Being Published? A Systematic Review of Reasons Given by Investigators
Source: PLoS One. 2014 Oct 15;9(10):e110418. doi: 10.1371/journal.pone.0110418 (PMC4198242; doi:10.1371/journal.pone.0110418)
Supplement: Appendix S2 — The main characteristics of reports that provided data on reasons for non-publication of studies. (PDF) [file pone.0110418.s002.pdf]

Appendix S2: The main characteristics of reports that provided data on reasons for non-publication of studies

Study	Source of studies	Types of studies	Survey method	Year of survey	Definition of non-publication	No. of not published / not submitted	Reasons for non-publication	Notes
**Ammenwerth & de Keizer (2007)**	A survey of 722 academics. 118 respondents revealed 217 evaluation studies of which 107 were unpublished or only published in internal reports or local publications.	Evaluation studies of health care IT	A written, email-based survey. (Response rate 18.8%, 136/722)	2006	Unpublished or only published in internal reports or local publications	107 /NA	Not formally published:<br>-Planned or in preparation: around 1/3<br>-Not of interest for others: around 1/3<br>-No time for writing: around 1/5<br>-Limited scientific quality: around 1/6<br>-Political & legal reasons: 1/7<br>-Only meant for internal use: 1/7	Provided only crude results. Mentioned that only 3 respondents explicitly indicated lack of publication due to negative results.
**Balasubramanian et al (2006)**	Abstracts (n=241) presented at the annual meeting of the Association of Surgeons of Great Britain and Ireland in 1997.	Mixed: clinical and translational	Authors of unpublished studies were contacted by a postal questionnaire. (Response rate: 57.6%, 49/85)	2003	Not fully published in journals or in alternative media including theses and books	38/28	Reasons for non-submission:<br>-lack of time: 8/30<br>-low priority: 6/30<br>-preliminary work for a larger study: 6/30<br>-published studies with similar findings: 5/30<br>-co-investigators left: 7/30<br>-others' responsibility for writing: 4/30<br>-results not sufficiently novel: 4/30<br>-statistical analyses were inconclusive: 1/30<br>-a low likelihood that journals would accept it for publication because of methodological limitations: 8/30<br>-a low likelihood that journals would accept it for publication because of insufficient interest among readership: 2/30	Unclear about denominator (n=30) used in the report.
**Bullen & Reeve (2011)**	Dissertations of public health Masters from 1991 to 2004, at the University of Auckland	Mixed design/types	A survey of graduates by emails, or letters or telephone contacts. (Response rate 74%, 77/104)	2006	Not published in journals	NA /NA	Reported barriers to writing an article:<br>-lack of time 63% (30/47)<br>-lack of support from staff 35%<br>-low confidence in ability to write 29%<br>-findings not being novel enough 36% (17/47)	

Study	Source of studies	Types of studies	Survey method	Year of survey	Definition of non-publication	No. of not published / not submitted	Reasons for non-publication	Notes
**Calnan et al (2006)**	A qualitative study of purposive sample of genetic scientists (n=6)	Genetic epidemiology	Qualitative face-to-face interview. (Response rate: not applicable)	Before 2006	N.A.	NA /NA	"Those pressured by both the continuing need to gain research grants and the UK universities' research assessment exercise felt that trying to publish negative results was a waste of scarce resources."	A qualitative, interesting study, without quantitative data.
**Camacho et al (2005)**	Abstracts (n=275) of phase I trials submitted to the annual meeting of the Am Society of Clinical Oncology in 1997	Phase I oncology clinical trials	Emailed a questionnaire to authors of abstracts if full articles were not identified. (Response rate 69.2%, 101/146)	2001	Not published in peer-reviewed journals	66 /55	Reasons for non-publication:<br>-lack of time: 23<br>-manuscript in preparation: 15<br>-manuscript submitted: 6<br>-rejection from journal: 5<br>-relocation of authors: 14<br>-incomplete study: 13<br>-results not interesting: 11<br>-not in sponsor's interest: 3<br>-conflict of interest: 1<br>-other: 2	65 authors of the unpublished abstracts provided 95 different reasons for not having pursued publication in peer reviewed journals.
**Canosa et al (2011)**	Abstracts (n=140), randomly selected out of 894 abstracts presented at the 33rd Argentinean Pediatric Congress (PAC) in 2003	Mixed	Questionnaires	2008-2010	Not fully published in journals	124 /117	Rejected by journals: 1.6% (n=124)<br>No reason given: 3.4% (n=124)<br>**Reason for non-submission (n=117):**<br>-lack of time: 35.9%<br>-methodological limitations: 15.4%<br>-not classifiable: 11.7%<br>-results not sufficiently novel: 7.7%<br>-publication not a concern: 7.7%<br>-published studies with similar findings: 6.8%<br>-lack of knowledge regarding publication process: 4.3%<br>-study conducted only for meeting presentation: 3.4%<br>-loss of contact with other members: 2.6%<br>-others' responsibility to write: 1.7%<br>-low priority of publication: 1.7%<br>-difficulty with co-authors: 0.8%<br>-study not completed: 0.3%	Study in Spanish. Google translate was used to get reasons for not publishing from Table 3.<br>Of the 124 unpublished work, 1.6% were rejected, 95% were not submitted for publication and 3.4% reported no reason.

**Study**	**Source of studies**	**Types of studies**	**Survey method**	**Year of survey**	**Definition of non-publication**	**No. of not published / not submitted**	**Reasons for non-publication**	**Notes**
**Cartwright et al (2007)**	Abstracts (n=130) presented at the International Continence Society Meeting in 2003	Mixed	Emailed a questionnaire to authors of abstracts if full articles were not identified. (Response rate 63.4, 26/41)	2006	Not published in peer-reviewed journals	25 /24	Rejected by journal: 1/25<br>Other reasons for non-publication:<br>-still in process of submission: 4 (17.4%)<br>-preliminary work for larger study: 6 (26.4%)<br>-lack of interest from journals: 7 (30.4%)<br>-no time: 6 (26.4%)<br>-responsibility lay elsewhere: 3 (13.0%)	Of 41 authors contacted, 26 responded regarding their unpublished abstracts. It is unclear why the percentages were calculated using 23 as the denominator.
**Cooper et al (1997)**	From 159 studies (by under graduates, post graduates, or faculty members) approved by the Department of Psychology Human Subjects Committee at a state university in 1986-1988	Mixed: class projects, theses or dissertations, independent projects, or pilot tests for larger studies	Telephone contact followed by a formal interview of faculty sponsors or investigators. (Response rate 73%, 33/45)	Before 1997	Not fully published in journals or book (or chapters)	64 /50	**Prepared study for journal publication:**<br>-Rejected by journal: 9<br>-under revision or to be submitted: 5<br>**Reasons for non-submission (n=50):**<br>-publication not an aim: 48%<br>-class project only: 30%<br>-assistant lost interest: 26%<br>-no significant results: 22%<br>-results were not interesting: 20%<br>-design or operational problems: 12%<br>-researcher did not recall: 6%<br>-others lost interest: 2%	Only included studies with written summaries (n=105). Investigators were allowed to give more than one reason.
**Dal-Re et al (2010)**	From studies (n=94) conducted by GlaxoSmithKline in Spain between 2001 and 2006	Clinical trials	Unclear	2009	Not published in peer-reviewed journals	37 /34	Under review: 2<br>Rejected by journal: 1<br>**Reasons for non-submission (n=34):**<br>-project cancelled: 16<br>-lack of time/resources: 12<br>-unknown: 6	Did not provide details on methods for data collection.
**De-Bellefeuille et al (1992)**	From 197 (randomly selected from 1058) abstracts included in the proceedings of the Am. Soc. Of Clin. Oncology in 1984	Mixed, including clinical trials	If the search failed to identify an article, a questionnaire was sent to authors. (Response rate 55%, 53/94)	Before 1992	Not published in peer-reviewed journals	41 /37	**Reasons for non-publication:**<br>-lack of time or other resources: 13<br>-insufficient priority: 9<br>-incomplete study: 5<br>-article not accepted for publication: 4<br>-modification after abstract submission: 1<br>-other: 12	44 total reasons from 41 respondents

**Study**	**Source of studies**	**Types of studies**	**Survey method**	**Year of survey**	**Definition of non-publication**	**No. of not published / not submitted**	**Reasons for non-publication**	**Notes**
**Decullier et al (2005)**	From 982 research protocols (649 with follow up information) approved by French research ethics committee in 1994.	Mixed: descriptive, observational, experimental	A mailed questionnaire was sent to the PI of approved protocols. (Response rate 68.9%, 677/982)	2002	Not published as a scientific paper in peer-reviewed journals	102 /79	**Writing or submission in progress: 23/102**<br>**Not submitted (n=79):**<br>- negative results: 27<br>-published in other forms: 23<br>-paper rejected: 5<br>-other reasons: 17<br>-not available: 7	
**Decullier & Chapuis (2006)**	From 142 study protocols submitted for funding to the Greater Lyon region scientific committee in 1997.	Mixed: descriptive, experimental, analytical, clinical and non-clinical	A mailed questionnaire was sent to the PI of each submitted protocol. (Response rate 80%, 114/142)	2003	Not published as a scientific paper	17 /6	**In preparation or submitted:**<br>-papers in preparation or submitted: 11<br>**Not submitted (n=6):**<br>-lack of time to write up: 2<br>-results not interesting enough: 1<br>-unknown or blank: 3	Only 51 completed studies.<br>Not able to separate submitted from in-preparation.
**Dickersin et al (1987)**	A survey of 318 authors who had published trials in 1980-1981.	Clinical trials	Mailed questionnaires to authors of RCTs. (Response rate 66.7%, 212/318)	1981	Not defined, and subject to investigators' interpretation	204 /176	**Submitted but not accepted: 23/204**<br>-in peer review: 10<br>-rejected by journal: 13<br>**Did not submit: 176/204**<br>-paper in preparation: 15<br>-results negative: 51<br>-lack of interest: 22<br>-sample size problem: 23<br>-poor methodology: 8<br>-side effects: 13<br>-external group problem: 10<br>-controversy: 3<br>-unknown or blank: 31	Did not submit' includes 74 RCT stopped
**Dickersin et al (1992)**	From a total of 737 studies approved in 1980 or prior to 1980 by 2 IRBs that serve the Johns Hopkins Health Institutions.	Mixed: observational, clinical trials, other experimental	Interview of investigators. (Response rate 92.7%, 683/737 eligible studies)	1988	Studies were not reported in journal articles, monographs, books or chapters in books	124 /118	Manuscript rejected by journal: 6/124<br>Did not submit manuscript: 118<br>-Results not interesting: 37<br>-Design or operational problems: 40<br>-publication not an aim: 16<br>-other reasons: 25	

Study	Source of studies	Types of studies	Survey method	Year of survey	Definition of non-publication	No. of not published / not submitted	Reasons for non-publication	Notes
**Dickersin & Min (1993)**	From 293 trials funded by the National Institutes of Health in 1979 (198 studies that analysed data and with publication information)	Clinical trials	Interview of investigators (Response rate 74.1%, 217/293)	1988	Not published in journals	**14 /NA**	**Why not published:**<br>-results not interesting or no time: 42.8%<br>-co-investigator or other operational problems: 37.5%<br>-incomplete data analysis: 14.3%<br>-did not know the reason: **1/14**	The number of respondents was unclear. "The majority (63%) of investigators stated that they had not published their findings either because the trial or analysis was incomplete or because the results were not interesting."
**Donaldson & Cresswell (1996)**	Any work during training and submissions made for the Part II examination for Membership of the Faculty of Public Health Medicine, by public health medicine trainees during 1974-1994	Mixed	A postal questionnaire survey. (Response rate 75%, 38/51)	1995	Not published in peer-reviewed journals	NA /NA	**Barriers to publication:**<br>-publication not a priority by the training department: 30 (79%)<br>-lack of time: 25 (66%)<br>-lack of mentor/co-author: 22 (58%)<br>-local relevance only: 11 (29%)<br>-lack of individual skill: 9 (24%)<br>-lack of personal motivation: 6 (16%)<br>-moving from post to post: 3 (8%)<br>-could not have published more: 2 (5%)	38 respondents identified barriers which they considered had reduced their capacity to publish their work.
**Drury et al (2012)**	Abstracts (n=282) presented at annual meeting of the Society for Cardiothoracic Surgery in GB & Ireland from 2003-2007	Mixed	If no article was identified on MEDLINE, authors were contacted through an email questionnaire. (Response rate 53.3%, 48/90)	2010	Not published in peer-reviewed journals	48 /41	**Submitted but rejected: 7 (14.6%)**<br>**Never submitted: 41 (85.4%):**<br>-low priority: 29.6%<br>-low likelihood of acceptance: 24.1%<br>-study ongoing and difficulties with co-authors: 14.8%	The denominator used for the reported percentages was unclear (unlikely to be 48 or 41).
**Dyson & Sparling (2006)**	283 abstracts presented at Am. College of Veterinary Anesthesiologists during the years 1990 through 1999.	Mixed design/types	Email messages sent to the authors of the unpublished studies. (Response rate 73%)	2003	Not fully published in journals or book (or chapters)	55 /NA	**Common reasons for not publishing**:<br>-too little time<br>-more interest in carrying out the work than in writing it up<br>-other more demanding tasks<br>**Rare reasons for not publishing:**<br>-rejection by the 1st journal submitted to<br>-project contained insufficient data or value<br>-refusal of permission by a private funding agency<br>**In the process of writing up: 10%**	Did not provide detailed data on reasons.

Study	Source of studies	Types of studies	Survey method	Year of survey	Definition of non-publication	No. of not published / not submitted	Reasons for non-publication	Notes
**Easterbrook & Berlin (1991)**	From 487 projects approved by the Central Oxford REC between 1984 and 1987.	Mixed design/types	Telephone interview or postal questionnaire. (response rate 92%, 487/530)	1990	Not fully published in journals	78 /43	**Submitted or published elsewhere 35**<br>**Other reasons**:<br>-null results 26<br>-methodology limitation/logistic problem 21<br>-sponsor control of data 19<br>-analysis incomplete 19<br>-manuscript rejected 16<br>-publication not aim of study 13<br>-too busy or lost interest 11<br>-unimportant results 10<br>-co-investigator left 5	Investigators of 29 studies cited 2 or more reasons.
**Green & Mar (2006)**	Manuscripts withdrawn or rejected, from 123 research papers submitted to Australian Family Physician between 2002 and 2004.	Mixed	Emailed a survey of 8 standard questions to authors. (Response rate 41%, 50/123)	2005	Not published in journals after being withdrawn or rejected	8 /NA	The primary reason for authors withdrawing a paper, or failing to resubmit, was being too busy.	Did not provide detailed data on reasons.
**Hartling et al (2004)**	From 166 RCTs (out of 393) presented at the Society for Pediatric Research meetings from 1992 to 1995.	Clinical trials (RCTs)	Mailed questionnaires. (Response rate: 38% for unpublished vs 56% for published studies)	2000	Not fully published in journals	47 /39	**Had submitted: 8/47**<br>**Non-submission: 39/47:**<br>-not enough time (n=39) 56.4%<br>-trouble with coauthors (n=38) 28.9%<br>-thought that journal was unlikely to accept (n=38) 26.3%<br>-results were not statistically significant (n=38) 23.7%<br>-results were not important enough (n=38) 18.4%<br>-other papers already published with similar findings (n=38) 15.8%<br>-study quality poor (n=37) 13.5%<br>-not worth the trouble (n=37) 10.8%<br>-results did not support the hypothesis (n=38) 5.3%	% cited as important.

Study	Source of studies	Types of studies	Survey method	Year of survey	Definition of non-publication	No. of not published / not submitted	Reasons for non-publication	Notes
**Harvey et al (2010)**	From 442 abstracts (by 396 authors) presented at the annual meetings of the Medical Library Association for the years of 2002 and 2003.	Mixed design/types	Online Survey Monkey questionnaire. (Response rate 44.8%, 155/346)	2008	Not fully published in peer-reviewed or non-peer-reviewed journals	114 /111	Submitted: 3/114<br>No submission: Main, (n=112) Second (n=82)<br>-wish to expand the study 8, 20<br>-methodological problems 2, 11<br>-fear of rejection 2, 5<br>-time restriction 49, 23<br>-post change 9, 5<br>-not suitable for publication 19, 4<br>-never intended publication 10, --<br>-lost interest 3, --<br>-lost support 2, --<br>-moved to other projects 1, 5<br>-miscellaneous 7, 6<br>-no other reason n.a., 7	Investigators were asked to select the main reasons they did not submit articles for publication, and next asked to choose any secondary reasons.<br>Number of unsubmitted studies was not explicitly reported.
**Hashkens & Uziel (2003)**	From 257 abstracts submitted to 4th Park City Pediatric Rheumatology meeting in 1998.	Mixed design/types	Questionnaires sent to authors of abstracts if no article was identified in the MEDLINE search. (Response rate 66%, 109/165)	2002	Not published in peer-reviewed journals	109 /97	**Submitted: 12/109**<br>-rejected by journal: 6<br>**Non-submission: 97/109**<br>-case report: 8<br>-previously reported: 5<br>-non-positive results: 2<br>-methodological problems: 2<br>-desire to expand paper: 42<br>-low priority or lack of time: 47<br>-fear of rejection: 13<br>-author moved or passed away: 4<br>-no decision on journal: 1	More than 1 reason could be given.

Study	Source of studies	Types of studies	Survey method	Year of survey	Definition of non-publication	No. of not published / not submitted	Reasons for non-publication	Notes
**Hoeg et al (2009)**	From 559 abstracts presented at the annual meetings of the Am. Society of Clinical Oncology in 1997, 1999 and 2001.	Phase II oncology clinical trials	Contacting authors by email if no article was identified. (Response rate 36%, 71/198)	2006	Not fully published in peer-reviewed journals	71 /NA	Reasons for failure to publish<br>-results not of interest 24<br>-lack of time 22<br>-relocation of authors 16<br>-study not completed 10<br>-not in sponsor's interest 8<br>-publication in non-peer reviewed journal 5<br>-manuscript in progress or under review 4<br>-drug development discontinued 3<br>-manuscript rejected 3<br>-disagreement among authors 1<br>-awaiting longer follow up 1<br>-study in progress 1<br>-conflict of interest 1	Authors may have provided more than one reason for non-publication.
**Hopewell & Clarke (2001)**	From 87 abstracts of methodological research presented at Symposium of Systematic Reviews in 1998 or Cochrane Colloquium in 1995.	Methodological research	A letter and a brief questionnaire sent to the contact authors. (Response rate 95.6%)	2000-2001	Not fully published in journals	37 /36	Reasons for not publishing:<br>-low priority or too busy 9<br>-not deemed appropriate 7<br>-findings became outdated 2<br>**-rejected by journal 1**<br>-subject area too specific 1<br>-internal Cochrane issue 1<br>-concerns over unity of approach 1<br>-unknown 15	Unpublished studies do not include 11 studies that authors plan to publish. One reason for each unpublished study. 22 of the 37 authors of the unpublished research provided a reason.
**Hosking & Albert (2002)**	98 UK specialist registrars and consultants within 5 years of appointment at a large district general hospital.	Mixed design/types	A questionnaire was sent to doctors about their "bottom drawer papers". (Response rate 62%)	2001	Not published in peer-reviewed journals	64 /52	Reasons for bottom drawer papers (5 most common) of 124 reasons provided overall:<br>-change of priorities and decision to spend time on other projects 32 (26%)<br>-move to another hospital 23 (18.5%)<br>-paper was rejected and I gave up 12 (10%)<br>-lack of support to the writing process 11 (9%)<br>-other 14 (11%)	Denominator for the reported percentages is n=124, which seemed to be the total number of reported reasons.

**Study**	**Source of studies**	**Types of studies**	**Survey method**	**Year of survey**	**Definition of non-publication**	**No. of not published / not submitted**	**Reasons for non-publication**	**Notes**
**Krzyzanowska et al (2003)**	From 510 abstracts presented at the Am. Society of Clinical Oncology between 1989 and 1998.	Large, phase 3, RCTs (n≥200)	If no publication identified by searching, authors were contacted by email or regular mail. (Response rate 53.5%, 54/101)	2001-2002	Not fully published	34 /25	Reasons for lack of publication:<br>-lack of time, funds or other resources 14<br>-study incomplete with intent to publish 6<br>-article submitted but not accepted 5<br>-manuscript in preparation 5<br>-manuscript under review 4<br>-insufficient priority to warrant publication 4<br>-other 5<br>-reason not provided 6	34 of the 40 authors of unpublished trials provided a reason regarding lack of publication. Some authors provided more than 1 reason. No. of not submitted is estimated by removing studies rejected or under review.
**Misakian & Bero (1998)**	Studies funded between 1981 and 1995, identified by information obtained from 76 of 89 organizations contacted that potentially funded research on passive smoking.	Mixed design/types, studies of passive smoking	Semi-structured telephone interviews of principal investigators. (Response rate 83%, 65/78)	1996-1997	Not fully published in journals (some results were not in-press nor published in a peer- or non-peer-reviewed journal)	59 /56	Reasons for not publishing (of 59 studies with some unpublished results):<br>-ongoing data collection/analysis 33<br>-lack of time 26<br>-competing priorities (such as recently funded studies, passive smoking only being a minor component or career changes) 1 1<br>-nonsignificant results 2<br>-rejected from a journal, but being resubmitted 3 (studies)	The 3 rejected studies (4 papers) were re-submitted.
**Montane & Vidal (2007)**	248 abstracts presented at the Congresses of the Spanish Society of Clinical Pharmacology in 1994, 1996, and 1998.	Mixed design/types	Authors were contacted by emails. (Response rate 65%, 66/102)	2003-2004	Not fully published in journals	97 /89	Reasons for not publishing (of 97 abstracts):<br>-lack of time 38 (39%)<br>-not interesting for publication 33 (34%)<br>**-submitted and rejected 4 (4.1%)**<br>-lack of authors' coordination 4 (4.1%)<br>-study not finished 3 (3.1%)<br>-unknown or not remembered 7 (7%)<br>-other (not specified) 8 (8%)<br>**Total 97 (100%)**	It seems one reason for each unpublished study (as authors had to choose 1 reason from a list).

Study	Source of studies	Types of studies	Survey method	Year of survey	Definition of non-publication	No. of not published / not submitted	Reasons for non-publication	Notes
**Morris et al (2011)**	A study of factors associated with the publication of scholarly articles by pharmacists who had published at least one article in 2008	Mixed, scholarly articles	A web-based survey. (Response rate 73%)	2009	N.A.	NA /NA	**Importance of barriers to the publication Mode score (% respondents):**<br>-lack of time 5 (56.3%)<br>-lack of collaboration 4 (32.9%)<br>-lack of knowledge about publication process 3 (25.0%)<br>-lack of resources 3 (25.7%)<br>-lack of encouragement 3 (33.5%)<br>-lack of incentives 3 (28.3%)<br>-not emphasized at institution 3 (25.8%)<br>-rejection of manuscript for publication 3 (32.2%)<br>-lack of confidence in research ability 3 (28.6%)<br>-lack of confidence in writing ability 3 (27.1%)<br>-lack of leadership skills 3 (27.0%)<br>-fear of rejection 1 (39.2%)<br>-not included in job description 1 (28.0%)	Mode score: 1 -not important, 3 -somewhat important, 5- very important.
**Okike et al (2012)**	918 manuscripts rejected by the Journal of Bone and Joint Surgery (American Volume) between 2004 and 2005.	Mixed design/types	A survey was emailed to authors of rejected manuscripts. (Response rate 55%, 507/918)	2010	Not published in journals	74 /43	**Reasons for non-publication (n=74):**<br>-lack of time 38 (51%)<br>-lack of interest by journals 17 (23%)<br>-other papers with similar findings already published 9 (12%)<br>-difficulty with co-authors 7 (10%)<br>-publication not worth the trouble 7 (10%)<br>-results not important enough 7 (10%)<br>-lack of funds or other resources 7 (10%)<br>-poor methodology 4 (5%)<br>-additional data collection 4 (5%)<br>-statistical analysis not positive 0<br>-other 12 (16%)	Several authors gave multiple reasons for non-publication.
**Oliveira et al (2009)**	From 313 orally presented abstracts at the Urological Brazilian Meeting in 2003.	Mixed design/types	Email and telephone contact of authors of unpublished studies. (Response rate 80.5%, 154/191)	2008	Not fully published in journals	154 /131	Reasons for lack of publication:<br>-no attempt to publish 57% (88)<br>-awaiting a larger sample 16% (25)<br>-under review by journal 11% (17)<br>-responsibility of another author 7% (11)<br>-other 7% (11)<br>**-study rejected 4% (6)**	No. of authors who responded was estimated (191 x0.805).

Study	Source of studies	Types of studies	Survey method	Year of survey	Definition of non-publication	No. of not published / not submitted	Reasons for non-publication	Notes
**Perez et al (2004)**	Abstracts (n=79) presented at the 1998-2000 annual scientific meetings of the Israel Society of Rheumatology.	Mixed design/types	A survey of authors of abstracts no found by searching MEDLINE. (Response rate 100%, 16/16)	2002	Not fully published in peer reviewed journals	16 /16	The main cause cited by authors for not publishing was lack of time to prepare manuscripts or a desire to further expand the study. 11/16 authors still plan to submit a paper.	Published in Hebrew. Information obtained from the Abstract. Not able to use Google Translate.
**Petticrew et al (2007)**	Abstracts (224) presented at British Sociological Association Medical Sociology meetings in 1998 and 1999.	Qualitative research studies	Authors of the unpublished studies were contacted. (Response rate: 56%, 125/224)	2006	Not fully published	125 /NA	A few respondents gave reasons for non-publication (though this did not appear to have been asked systematically). These included lack of time and job moves. Also reported that conference presentation aimed mainly to discuss with peers rather than as a prelude to publication. One reported that they had 'lost heart' after poor reviews.	Did not provide further data on reasons.
**Reveiz et al (2006)**	From a random sample of 525 authors of RCTs identified by searching MEDLINE from 1999 to 2003.	RCTs	A questionnaire was emailed to authors. (Response rate 7.6%, 40/525)	2004	Not published in journals	10 /NA	The most frequently given reasons for not publishing were: lack of time for completing the analysis and preparing the manuscript; contractual obligations with the pharmaceutical industry; lack of statistical power; methodological errors in designing; editorial rejection; material was the subject of an undergraduate thesis; and loss of information	Did not provide further data on reasons.
**Reysen (2006)**	A survey of 236 (of 1359 potential participants, 17%) psychology department faculty regarding their opinions about publication of non-significant results.	Studies with non-significant results	Email survey (Response rate 17%, 236/1359)	2004	N.A.	NA /NA	Reasons for not writing a manuscript (aside from time constraints or little chance of publication) of 128 respondents included flawed methods or design (19 people, 15%), the results were not meaningful or would not advance the literature (9), could not pinpoint the reasons for non-significance (5), bad study (2).	Further data available on a 9-point rating scale (1 -strongly disagree, 9-strongly agree).
**Rodriguez et al (2009)**	Research protocols (n=190) submitted to a Hospital IRB (2001-2006): 84 studies were conducted and 40 published.	Mixed	A questionnaire was administered (Response rate: NR)	2008	N.A.	44 /NA	The main cause for not publishing was the lack of time for the writing of the article.	No further data provided. Published in Spanish, and used Google Translate to obtain information.

Study	Source of studies	Types of studies	Survey method	Year of survey	Definition of non-publication	No. of not published / not submitted	Reasons for non-publication	Notes
**Rotton et al (1995)**	A survey of 740 US authors of empirical articles published in 75 psychology journals in Oct 1984 to January 1985.	Empirical research studies	A letter with an anonymous questionnaire. (Response rate 63% or 478 replies)	Before 1995	Not fully published	NA /NA	Reasons for deciding against publication (authors could choose as many pre-specified answers as they liked when considering the last paper they filed rather than publishing, n=336):<br>-failure to replicate 4.8%<br>-unfavourable reviews 33.3%<br>-non-hypothesized results 4.5%<br>-inexplicable results 22.3%<br>-non-significance 59.9%	Authors have 'filed away' 15.4% of their work. If a manuscript was rejected, 93.3% of authors said they would have sent it to another refereed journal.
**Sanossian et al (2006)**	353 abstracts (108 oral presentations, 245 posters) presented at the International Stroke Conference in 2000.	Mixed	A questionnaire was emailed to authors of studies without evidence of publication. (Response rate 66.8%, 105/157)	2005	Not fully published in journals	74 /54	Submitted:<br>-Submitted and rejected 12 (16%)<br>-In various stages of submission 8 (11%)<br>Reasons for non-publication (n=74):<br>-no time 28 (37.8%)<br>-low priority 11 (14.8%)<br>-co-author responsibility/lack of participation 10 (13.5%)<br>-study ongoing 8 (10.8%)<br>-methodological limitations 6 (8.1%)<br>-different version published 3 (4.1%)<br>-other similar articles published 2 (2.7%)<br>-does not recall 1 (1.3%)<br>-no reason given 5 (6.7%)	
**Smith et al (2009)**	A survey of 5 years of graduates (2000-2004) from the Michigan State University Faculty Development Fellowship Program (n=90), to investigate factors related to publication of their fellowship research projects.	Mixed, scholarly articles	A survey via email on Survey Monkey. (Response rate 70%, 63/90)	2007	Not published in journals	NA /NA	Barriers to publication (n=50 respondents), several reasons allowed:<br>-lack of time 18 (36%)<br>-unable to complete project 11 (22%)<br>-paper rejected 8 (16%)<br>-lack of help 8 (16%)<br>-poor quality project 4 (8%)<br>-lazy not motivated 4 (8%)<br>-left position 2 (4%)<br>-not personal goal to publish 2 (4%)<br>-ongoing project 2 (4%)<br>-lacked confidence 2 (4%)<br>-change in paradigm 1 (2%)	Respondents could make more than one comment. Facilitators to publishing were stated as home/fellowship mentor (3), instruction during fellowship (3), gained confidence (1), experience of presenting poster (1), and "ton of work and a little luck" (1)

Study	Source of studies	Types of studies	Survey method	Year of survey	Definition of non-publication	No. of not published / not submitted	Reasons for non-publication	Notes
**Snedeker et al (2010)**	149 abstracts of studies from proceedings of 10 conferences between 1995 and 2004.	Studies of pre-harvest or interventions against foodborne pathogens	Email contact with authors of abstracts where a peer-reviewed full paper could not be located. (Response rate 43%, 34/79)	2009	Not fully published in peer-reviewed journals	34 /NA	Reasons for nonpublication (from 34 authors):<br>-lack of time and resources (often as had moved to a different position or institution) 13 (38%)<br>-peer reviewed publication not a priority (researchers in private industry or certain government agencies) (number not given)<br>-in the process of publication 3 (9%)<br>-published in non-peer reviewed reports or documents (number not given)	Insufficient data on reasons.
**Sprague et al (2003)**	465 abstracts presented at Annual Meetings of the Am. Academy of Orthopaedic Surgeons in 1996, of which 306 appeared unpublished according to Medline.	Mixed	Faxed or mailed a questionnaire to authors of abstracts for which a full paper was not located. (Response rate 65%, 199/306).	2002	Not fully published in journals	120 /71	**Under consideration by journal 14**<br>**Submitted and rejected 32**<br>**Reasons for non-submission for publication (of 71**):<br>-lack of time 33 (46%)<br>-study still ongoing 22 (31%)<br>-writing is a responsibility of a co-author 14 (20%)<br>-difficulty with co-authors 12 (17%)<br>-pursuit of publication low priority 9 (13%)<br>-low likelihood of acceptance due to methodology limitations 9 (13%)<br>-other papers with similar findings already published 3 (4%)<br>-plan to resubmit paper for publication 3 (4%)<br>-results not important enough 1 (1%)<br>-statistical analysis not positive 1 (1%)<br>-low likelihood of acceptance by journal because of insufficient interest to readers 1 (1%)<br>-different version of data published 1 (1%)<br>**Abstracts not recalled by investigato**r 3 (4%)	Respondents could make more than one comment.<br>A study focused on barriers and reasons.

Study	Source of studies	Types of studies	Survey method	Year of survey	Definition of non-publication	No. of not published / not submitted	Reasons for non-publication	Notes
**Stocker et al (2009)**	Abstracts (n=368) presented at the annual meeting of the German College of General Practitioners (DEGAM) in 1999-2003.	Mixed, general practice	Authors of abstracts that had not been published were surveyed (by telephone or email) (Response rate 84%, 148/177)	2007	Not fully published in a medical journal	113 /104	Reasons for non-publication (n=113)<br>-lack of time or overload 14 (12.4%)<br>-only for dissertation or thesis 14 (12.4%)<br>-poor study quality or subjects 13 (11.5%)<br>-difficulties with doctorial candidates 12 (10.6%)<br>-moved to other priorities 11 (9.7%)<br>-forgot/could not remember 10 (8.8%)<br>-daily working pressure 8 (7.1%)<br>-study not pursued 6 (5.3%)<br>-data collection is still ongoing 5 (4.4%)<br>-responsibility lies with other co-authors 5 (4.4%)<br>-submitted (under review) or planning to re-submit rejected manuscripts 3 (2.7%)<br>-accepted for publication, in print 3 (2.7%)<br>-rejected and no plan to re-submit 3 (2.7%)<br>-negative conference feedback 3 (2.7%)<br>-ignorance of publication opportunity 2 (1.8%)<br>-lack of financial incentive 1 (0.9%)	One reason for each unpublished study.<br><br>Published in German language. Used Google Translate to obtain information.
**ter Riet et al (2012)**	A survey of laboratory animal researchers in the Netherlands.	Animal research studies	Emails with a link to the internet-based questionnaire (SurveyMonkey). (Response rate 14-25% as 2000-3500 received the invitation, 474 respondents)	2011	Non-publication	NA /NA	**Causes of nonpublication (1=totally unimportant, 5=very important), n=444, medians (IQ range):**<br>-lack of statistically significant differences 4 (4-5)<br>-instrumentation/technical problems 4 (3-4)<br>-lack of time to write manuscripts 2 (2-3)<br>-loss of interest 2.5 (2-3)<br>-many studies are seen as pilot studies only 2 (2-3)<br>**Who are responsible for nonpublication:**<br>-senior researchers (supervisors) 4 (3-5)<br>-junior researchers 3 (2-4)<br>-editors 4 (3-4)<br>-reviewers/referees 4 (3-5)<br>-funders 4 (3-4)	Perceived importance score: 1-totally unimportant, 5-extremely important.<br>Estimated publication rate:<br>-not for profit institutes 80%<br>-for profit institutes 10%<br>-with large animals 90%<br>-with small animals 75%

Study	Source of studies	Types of studies	Survey method	Year of survey	Definition of non-publication	No. of not published / not submitted	Reasons for non-publication	Notes
**Timmer et al (2002)**	A random sample of abstracts submitted to the Digestive Diseases Week, a major gastroenterology meeting, between 1992 and 1995.	Mixed including controlled trial (326 abstracts), other clinical (336) and basic science research (174), total 836.	A mailed survey to all abstract authors.<br>(Response rate: 70% for authors of published abstracts and 56% for authors of unpublished abstracts)	2001	Not fully published	206 /156	Reasons for non completion or nonsubmission (n=112): Most important, Other<br>-lack of time 48, 76<br>-coinvestigator left 12, 32<br>-lack of interest 5, 25<br>-recruitment problems 7, 26<br>-methodology limitations 4, 21<br>-unimportant results 3, 16<br>-rejection anticipated 3, 10<br>-publication not the aim 3, 10<br>-negative results 3, 11<br>-external problems 6, 6<br>-side effects/ethical problems 1, 5<br>-equipment/software problems 2, 3<br>-no single decisive factor 15, --	Multiple responses were allowed for 'other factors'.
**Timmons & Park (2008)**	A qualitative study of a purposive sample of supervisors (n=10) and students (10 who published their dissertations and 10 who had not published) in a School of Nursing.	Research undertaken by nursing students	A telephone interview.<br>(Response rate: unclear)	2007	Non-publication	NA /NA	Students: For some students, publishing was not a priority. The dissertation as a means to a degree. Lack of encouragement from job managers. A few felt their work was not good enough to publish.<br>Supervisors' view: the graduates' demanding lives, shortage of time, the quality of the study, and students' enthusiasm.	A qualitative interview
**Tricco et al (2009)**	A survey of authors of a sample of systematic reviews published in 2005.	**Systematic reviews**	A survey administered through the Internet, fascimile, and postal mail.<br>(Response rate 56%, 348/625)	2006-2007	Not formally published	199 /NA	Main reasons for not publishing most recent SRs (n=52) and SRs in general (n=199):<br>-lack of time 23%, 31%<br>-rejected 19%, 17%<br>-other 13%, 16%<br>-SR too broad 12%, 6%<br>-In process 10%, 13%<br>-Few eligible SRs* 10%, 4%<br>* probably included studies?	A median of 2.0 unpublished SRs per reviewer (1-33). Also reported reasons for conducting SRs.

Study	Source of studies	Types of studies	Survey method	Year of survey	Definition of non-publication	No. of not published / not submitted	Reasons for non-publication	Notes
**Van Royen et al (2010)**	Abstracts presented at 10 European General Practice Research Network meetings between 1999 and 2006.	Mixed	Survey by email or postal questionnaire among all presenters. (Response rate 60%, 251/416)	2003, 2008	Not fully published in journals	138 /103	**The study is incomplete 37 (27%)**<br>Not started writing up yet 17 (12%)<br>In the process of writing up 31 (22%)<br>Manuscripts to be translated into English 9 (7%)<br>Paper ready & selecting journal to submit 9 (7%)<br>**Of those 35 submitted:**<br>Submitted and awaiting a response 10 (7%)<br>Submitted but rejected 16 (12%)<br>Resubmitting the same article 4 (3%)<br>Rewriting after it was rejected 3 (2%)<br>Resubmitted a revised version to a different journal 5 (4%)	Some respondents gave more than one answer.
**Vawdrey & Hripcsak (2013)**	62 clinical trials of electronic health records registered in clinicalTrials.gov	Clinical trials of electronic health records	PI were contacted if no publication was identified. (Response rate 47%, 7/15)	2012	Not published in journals	7 /NA	Reasons identified by PIs for the lack of publication: key members had left the project team, too busy or had not had sufficient time to publish, and manuscript rejected by journal editors (numbers of each response not provided)	Did not provide more details on reasons.
**Vuckovic-Dekic et al (2001)**	98 Serbian authors with abstracts presented at the 1st and 2nd Congress of the Balkan Union of Oncology in 1996 and 1998.	Mixed design/types	Authors contacted by a letter with a questionnaire. (Response rate 46%, 45 authors/98)	2001	Not fully published	21 /21	Reasons for failure to publish (of 21):<br>-not enough time 10 (48%)<br>-thought journals unlikely to accept 2 (10%)<br>-results not important enough 1 (5%)<br>-other papers with similar findings 1 (5%)<br>-too much trouble with coauthors 1 (5%)<br>-other reasons (including problems in design, sample size, financial problems etc) 6 (29%)	Other category includes 'problem in design, sample size, financial difficulties, etc'.

**Study**	**Source of studies**	**Types of studies**	**Survey method**	**Year of survey**	**Definition of non-publication**	**No. of not published / not submitted**	**Reasons for non-publication**	**Notes**
**Weber et al (1998)**	From 492 abstracts submitted to a meeting of the Society for Academic Emergency Medicine in 1991.	Mixed design/types	A questionnaire mailed to authors for whom no publication was found in the search.<br>(Response rate 84%, 223/266)	1996	Not fully published in peer-reviewed journals	223 /179	**Reasons for non-submission (authors asked to select a reason, presumably from a pre-specified list):**<br>-not enough time: 74 (42%)<br>-thought journals unlikely to accept: 35 (20%)<br>-results not important enough: 21 (12%)<br>-too much trouble with co-authors: 16 (9%)<br>-not worth the trouble: 13 (7%)<br>-other papers with similar findings: 11 (6%)<br>-statistical analysis not positive: 7 (4%)<br>-other reasons: 40 (22%)	
**Wong et al (2010)**	67 conference abstracts of RCTs included in systematic reviews of surgical treatments for benign prostatic enlargement.	RCTs	Questionnaires (with pre-paid return envelope) were mailed and also emailed to authors.<br>(Response rate 70%, 47/67)	2007	Not fully published	15 /14	**Reasons for non-submission (asked "what are the plans for full-text publication" offered the choices below (but conflict of interest added as a free text option):**<br>-being written up 8/14<br>-not written up due to lack of time 3/14<br>-not written up due to lack of funding 2/14<br>-conflict of interest 1/14<br>**Reasons for non-publication for submitted articles (could choose "not accepted by journal" or provide free text answer):**<br>-sponsoring commercial company did not wish to be fully published 1/15<br>Rejected due to methodological flaw 1/15	

Study	Source of studies	Types of studies	Survey method	Year of survey	Definition of non-publication	No. of not published / not submitted	Reasons for non-publication	Notes
**Woo et al (2012)**	Abstracts (n=2156) presented at the American College of Rheumatology and Association of Rheumatology Health Professionals Annual Meeting in 2006.	Mixed design/types	Authors of unpublished abstracts were surveyed via an internet questionnaire. (Response rate 14%, 65/459)	2011	Not published in peer reviewed journals (full-length articles or brief reports)	104 /NA	Main reasons for non-publication:<br>-insufficient time to prepare manuscript 35.2%<br>-co-author was responsible 29.6%<br>-the study was still ongoing 16.7%	Abstract only.<br>65 authors reflecting 117 abstracts.<br>Unclear about the denominator for the reported proportion.
**Woodrow et al (2012)**	Survey of 679 members of the International Society for Medical Publication Professionals (ISMPP), American Medical Writers Association, the Network Pharma community, and other groups.	Clinical trials	(Response rate 89%, 607/679)	2011	NA	NA /NA	Main reasons for non-publication of clinical trial data:<br>-compound discontinuation 40%<br>-journal rejection 36%<br>-poor trial design 31%<br>-damage to the product profile 27%	Abstract only.<br>1/3 completers aware of unpublished negative data from a clinical trial in the past 3 years.

Notes: NA – data not available
