# Supplementary material for: Why Are Medical and Health-Related Studies Not Being Published? A Systematic Review of Reasons Given by Investigators
Source: PLoS One. 2014 Oct 15;9(10):e110418. doi: 10.1371/journal.pone.0110418 (PMC4198242; doi:10.1371/journal.pone.0110418)
Supplement: Appendix S3 — Forest plots of stated reasons for non-publication of studies. (PDF) [file pone.0110418.s003.pdf]

Appendix S3: Forest plots of stated reasons for nonpublication of studies

Notes: The number of unpublished studies was used as the denominator to calculate the proportion of non-submission and specific reasons for non-publication for each included survey report. For estimating 95% confidence intervals, heterogeneity testing and meta-regression analyses, proportions of reasons were transformed to normally distributed values using the Freeman-Tukey transformation methods. Then the Freeman-Tukey transformed proportions were transformed back to usual proportions for the forest plots.

A3.1 – Proportion of non-submission in un-published studies

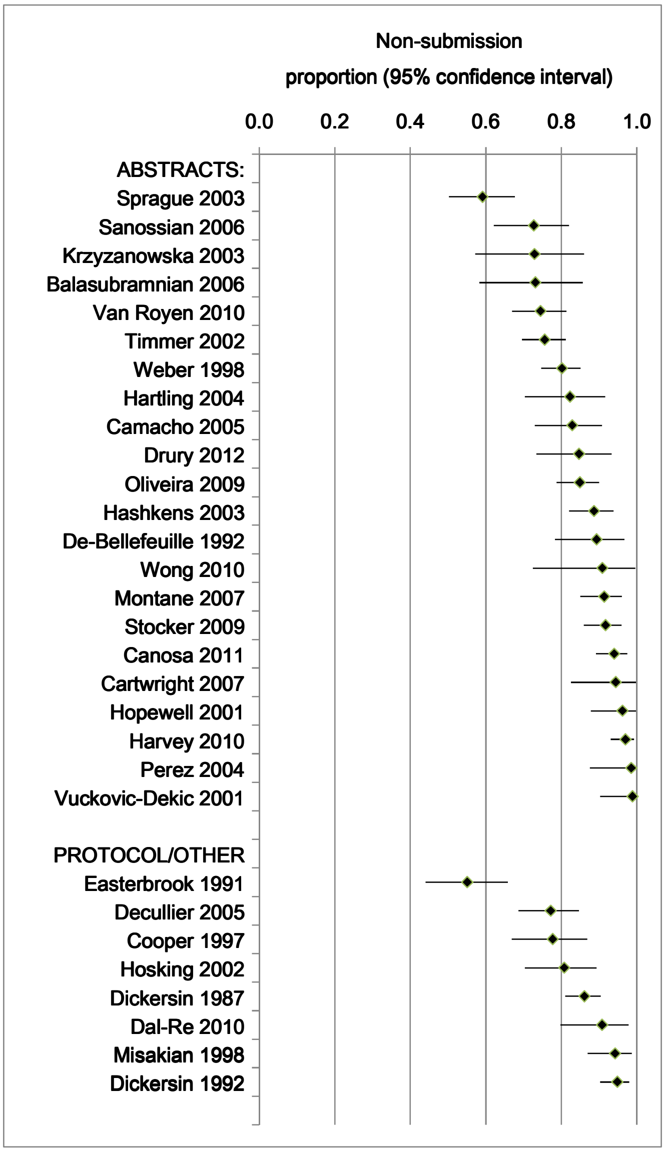

Heterogeneity test result: χ^2 =211.37, df=29; (P<0.001); I^2=86.3% (95% CI 81.5%, 89.8%)

A3.2 –Study incomplete or still ongoing

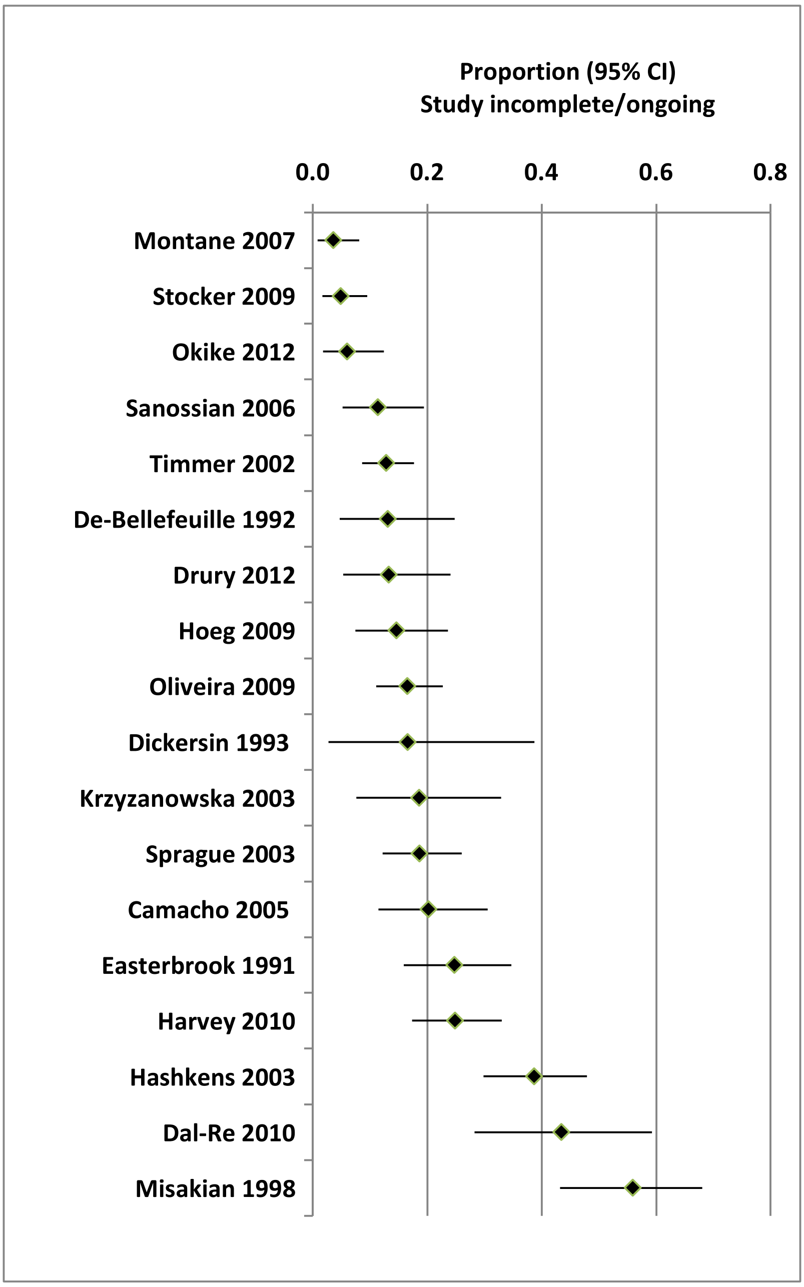

Heterogeneity test result: χ^2 =146.1, df=17; (P<0.001); I^2=88.4% (95% CI 83.1%, 92.0%)

A3.3 – Manuscript in preparation or under review

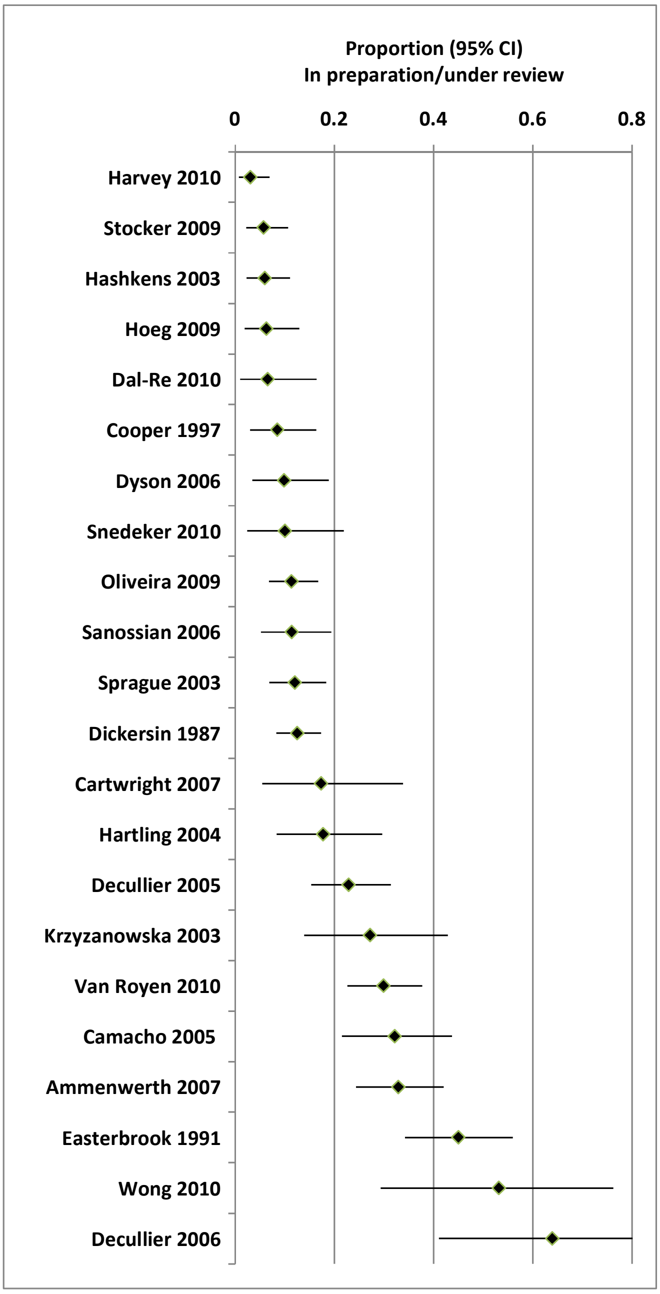

Heterogeneity test result: χ^2 =186.8, df=21; (P<0.001); I^2=88.8% (95% CI 84.3%, 91.9%)

A3.4 – Study not for publication (e.g., feasibility or pilot trials)

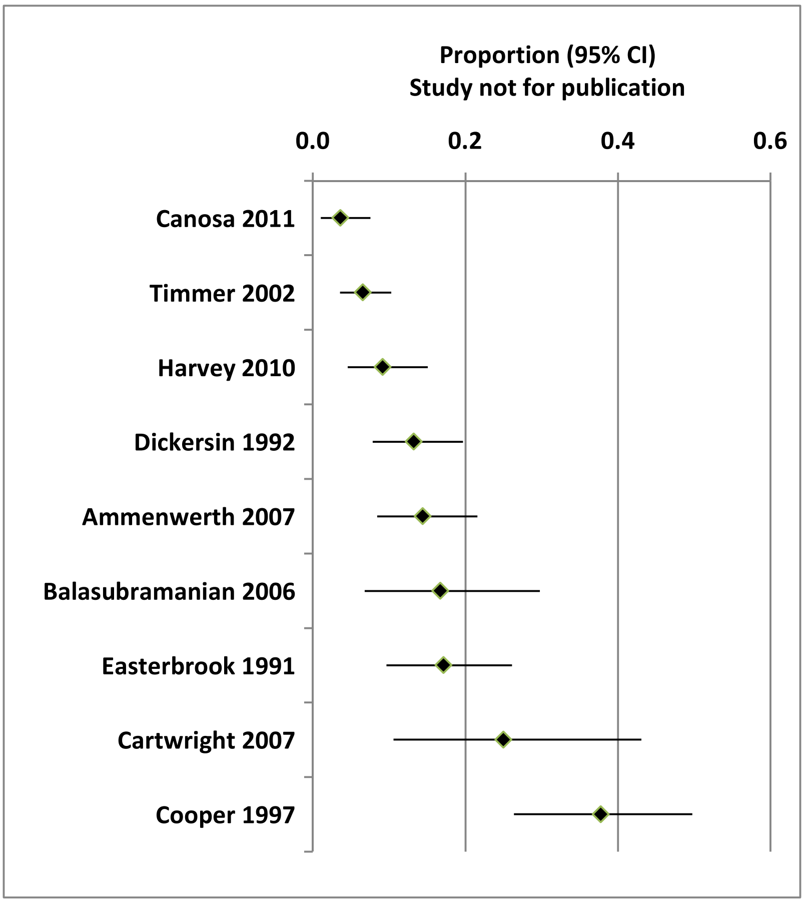

Heterogeneity test result: χ^2 =52.3, df=8; (P<0.001); I^2=84.7% (95% CI 72.7%, 91.4%)

A3.5 – Similar findings published by others

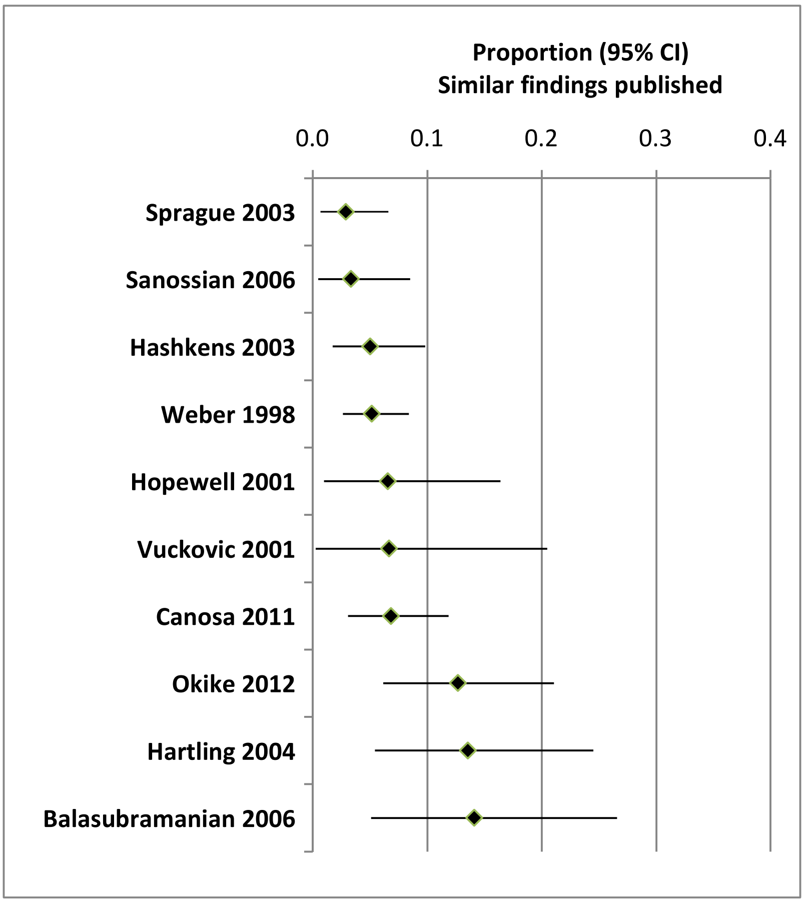

Heterogeneity test result: χ^2 =15.0, df=9; (P<0.091); I^2=40.0% (95% CI 0.0%, 71.4%)

A3.6 –Submission rejected by journal

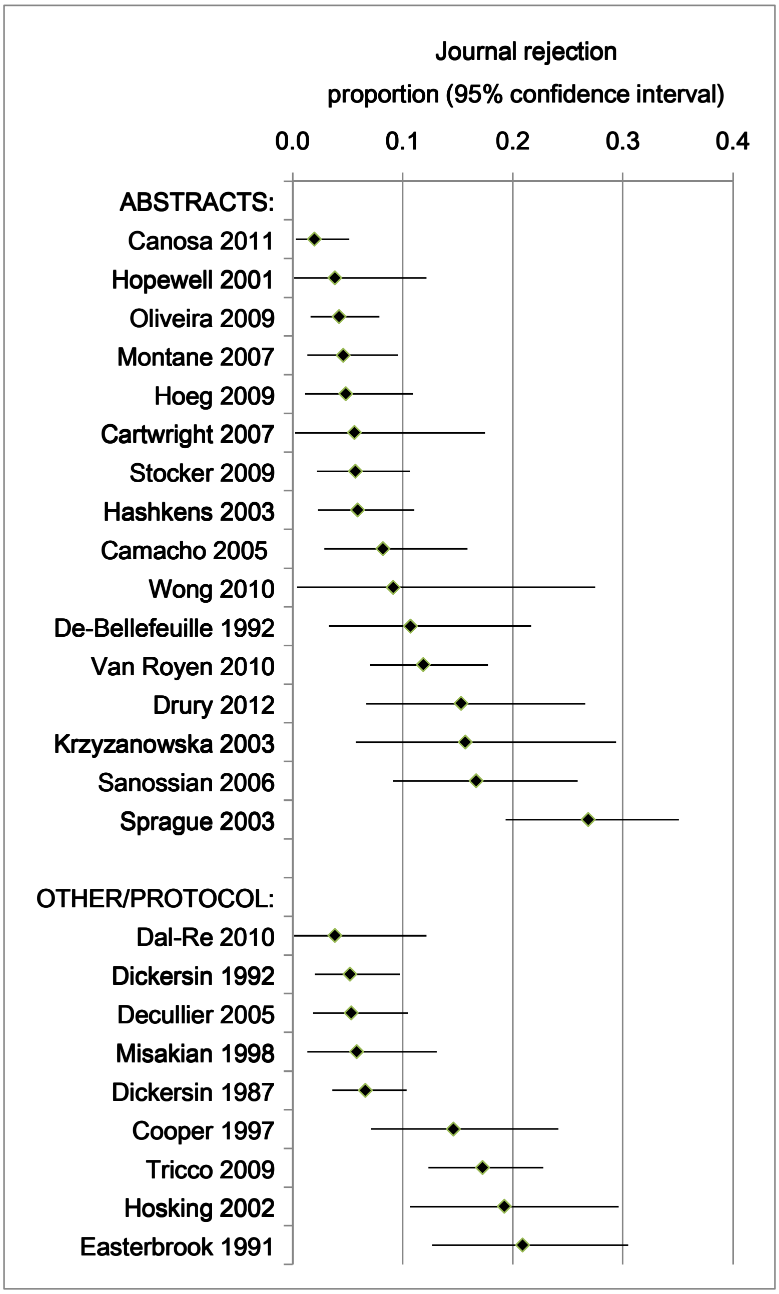

Heterogeneity test result: χ^2 =105.1, df=24; (P<0.001); I^2=77.2% (95% CI 66.7%, 84.4%)

A3.7 – Fear of being rejected

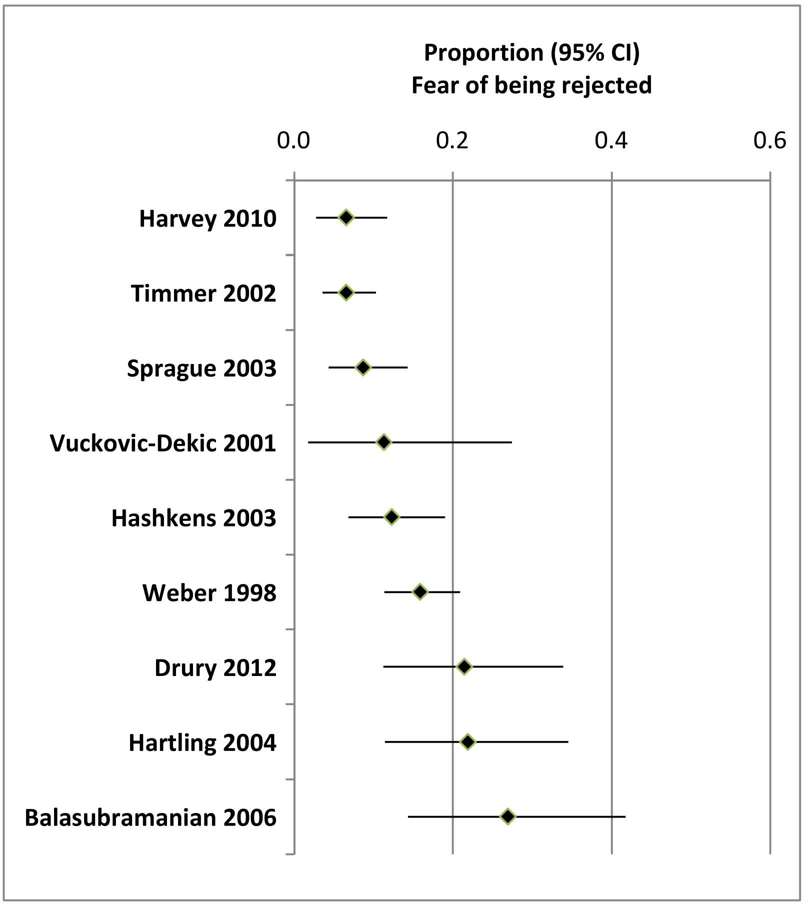

Heterogeneity test result: χ^2 =28.2, df=8; (P<0.001); I^2=71.7% (95% CI 44.2%, 85.6%)

A3.8 – Lack of time or low priority

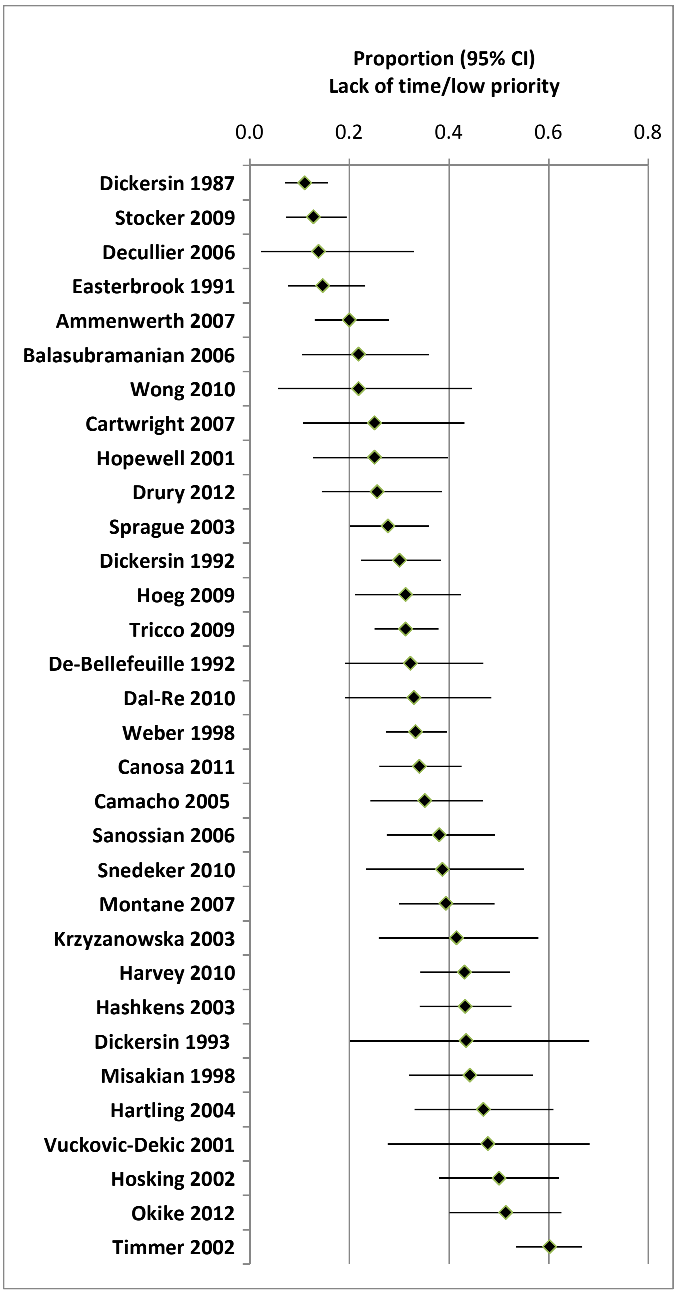

Heterogeneity test result: χ^2 =232.7, df=31; (P<0.001); I^2=86.7% (95% CI 82.3%, 90.0%)

A3. 9 – Not important or negative results

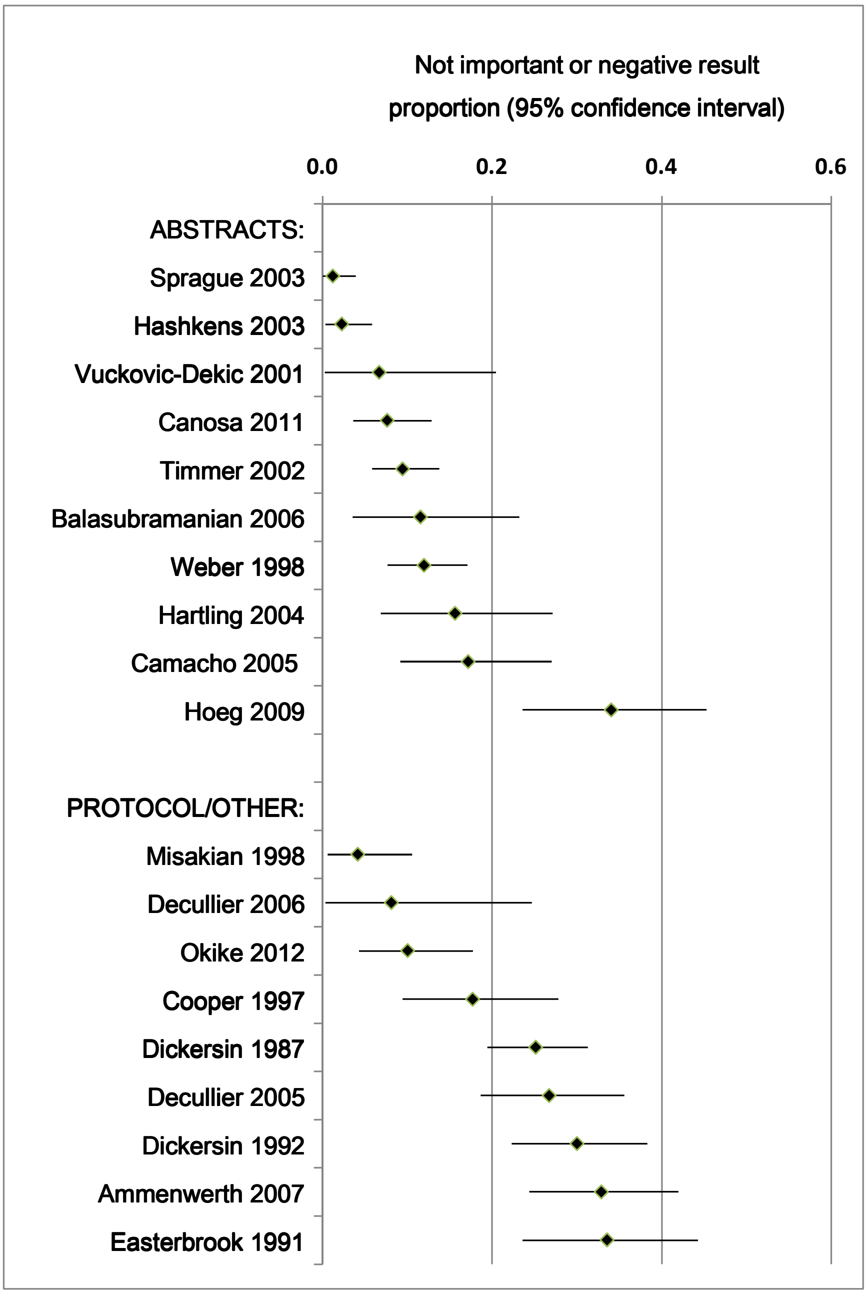

Heterogeneity test result: χ^2 =183.8, df=18; (P<0.001); I^2=90.2% (95% CI 86.2%, 93.1%)

A3.10 – Poor study quality or methodology

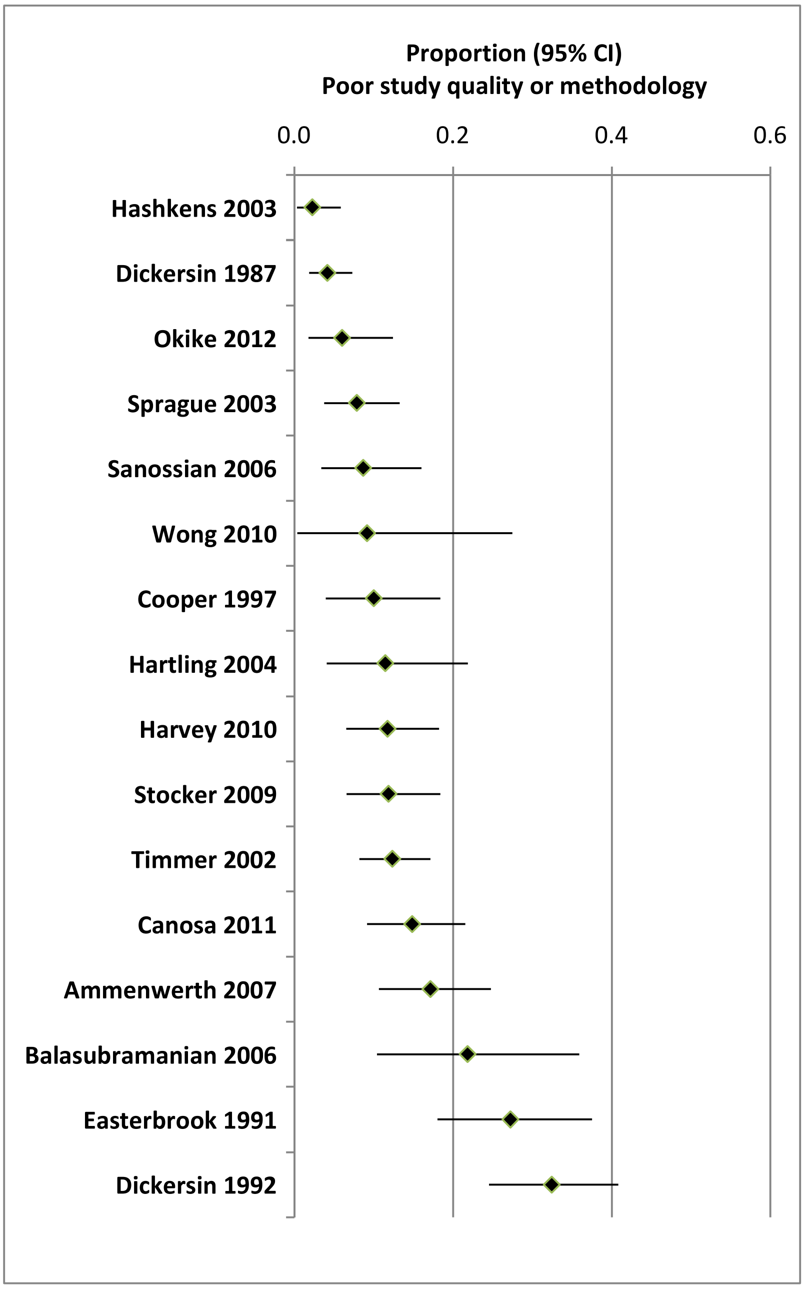

Heterogeneity test result: χ^2 =92.8, df=15; (P<0.001); I^2=83.8% (95% CI 75.1%, 89.5%)

A3.11 – Sponsor or funder problem

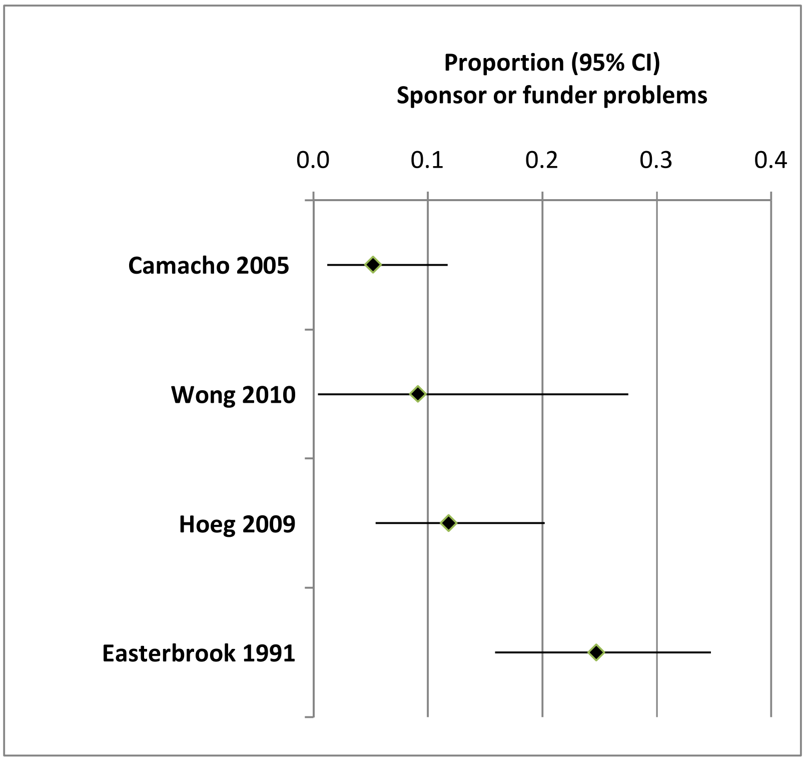

Heterogeneity test result: χ^2 =12.7, df=3; (P=0.005); I^2=76.4% (95% CI 35.3%, 91.4%)

A3.12 – Author/co-author problem

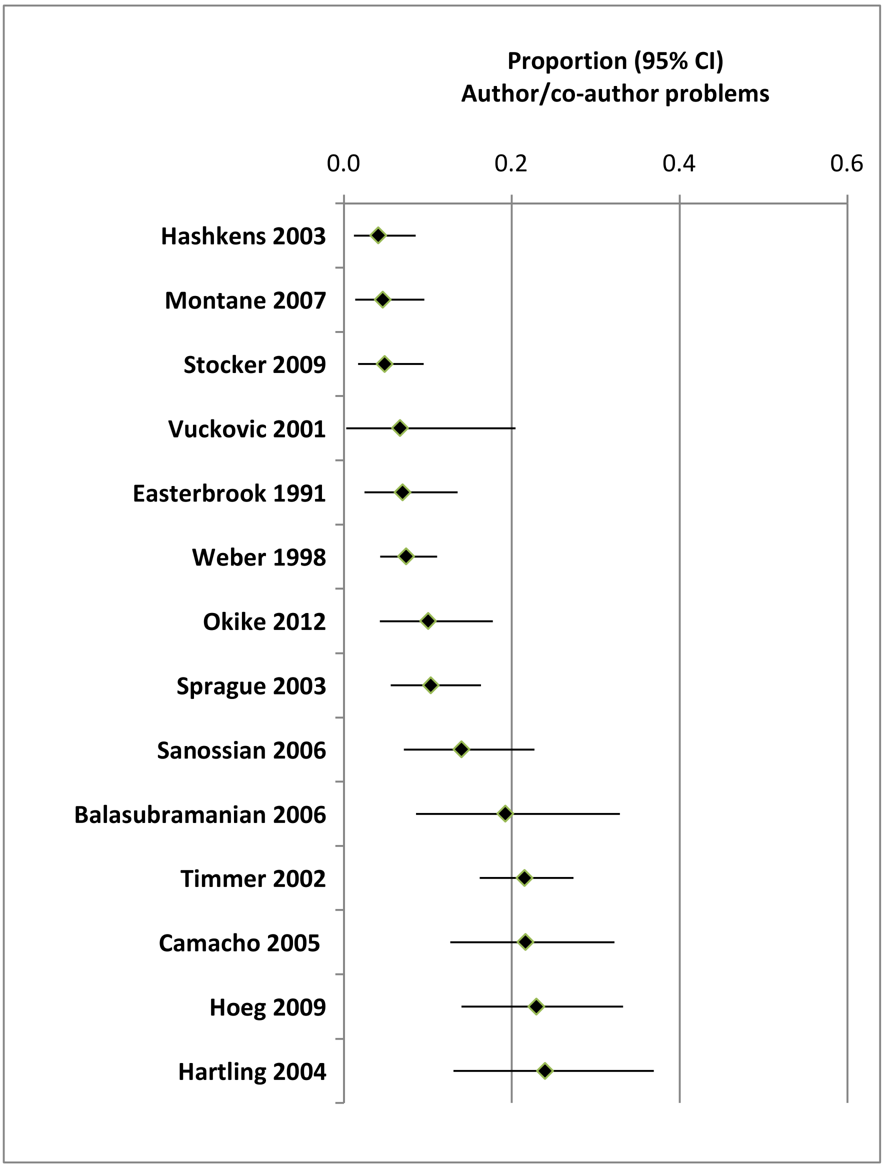

Heterogeneity test result: χ^2 =64.7, df=13; (P<0.001); I^2=79.9% (95% CI 67.1%, 87.7%)
