# Supplementary material for: Why Are Medical and Health-Related Studies Not Being Published? A Systematic Review of Reasons Given by Investigators
Source: PLoS One. 2014 Oct 15;9(10):e110418. doi: 10.1371/journal.pone.0110418 (PMC4198242; doi:10.1371/journal.pone.0110418)
Supplement: Appendix S4 — Results of meta-regression analyses of differences between subgroups. (PDF) [file pone.0110418.s004.pdf]

Appendix S4: Results of meta-regression analyses of differences between subgroups.

Reasons	No. of included surveys	Estimated coefficients (95% confidence interval); P-value				
		**Abstracts vs. other sources**	**Trials only vs. mixed designs**	**Surveys up to 2002 vs. since 2003**	**% Response rate**	**Rate of non-submission**
Non-submission	30	0.075 (-0.187, 0.337)<br>P=0.562	-0.032 (-0.334, 0.270)<br>P=0.831	0.107 (-0.130, 0.344)<br>P=0.363	0.216 (-0.579, 1.012)<br>P=0.581	
Study incompletion or ongoing	18	-0.304 (-0.672, 0.064)<br>P=0.099	0.129 (-0.268, 0.526)<br>P=0.501	-0.266 (-0.585, 0.053)<br>P=0.096	0.486 (-0.679, 1.650)<br>P=0.388	0.167 (-0.473, 0.808)<br>P=0.582
In preparation or under review	22	-0.249 (-0.608, 0.110)<br>P=0.164	0.069 (-0.312, 0.450)<br>P=0.710	-0.068 (-0.423, 0.288)<br>P=0.696	0.363 (-0.659, 1.384)<br>P=0.466	-0.537 (-1.088, 0.014)<br>P=0.055
Study not for publication	9	-0.274 (-0.678, 0.131)<br>P=0.154	N.A.	-0.137 (-0.594, 0.321)<br>P=0.502	0.271 (-0.707, 1.249)<br>P=0.523	-0.220 (-0.960, 0.519)<br>P=0.493
Similar findings published	10	-0.245 (-0.571, 0.081)<br>P=0.122	0.259 (-0.137, 0.655)<br>P=0.170	0.099 (-0.123, 0.321)<br>P=0.336	-0.517 (-1.300, 0.266)<br>P=0.162	0.145 (-0.159, 0.450)<br>P=0.296
Rejection by journal	25	-0.058 (-0.251, 0.136)<br>P=0.544	-0.082 (-0.310, 0.145)<br>P=0.462	-0.098 (-0.284, 0.087)<br>P=0.286	-0.321 (-0.991, 0.348)<br>P=0.330	-1.609 (-2.102, -1.115)<br>**P<0.001** [(1)]
Fear of being rejected	9	N.A.	0.258 (-0.289, 0.805)<br>P=0.301	0.112 (-0.256, 0.479)<br>P=0.496	0.019 (-1.285, 1.323)<br>P=0.973	-0.086 (-0.609, 0.438)<br>P=0.710
Lack of time or low priority	32	0.100 (-0.113, 0.313)<br>P=0.345	0.065 (-0.180, 0.311)<br>P=0.591	-0.112 (-0.316, 0.092)<br>P=0.271	-0.403 (-0.986, 0.181)<br>P=0.168	0.095 (-0.294, 0.485)<br>P=0.617

Reasons	No. of included surveys	Estimated coefficients (95% confidence interval); P-value				
		**Abstracts vs. other sources**	**Trials only vs. mixed designs**	**Surveys up to 2002 vs. since 2003**	**% Response rate**	**Rate of non-submission**
Not important or negative result	19	-0.288 (-0.576, 0.000) **P=0.050(2)**	0.267 (-0.109, 0.643) P=0.152	0.075 (-0.277, 0.428) P=0.657	-0.187 (-1.059, 0.686) P=0.656	-0.134 (-0.695, 0.426) P=0.613
Poor study quality or design	16	-0.123 (-0.395, 0.149) P=0.349	-0.191 (-0.546, 0.164) P=0.268	0.016 (-0.259, 0.290) P=0.902	0.309 (-0.436, 1.055) P=0.386	-0.036 (-0.499, 0.426) P=0.868
Sponsor/funder problems	4	-0.452 (-1.174, 0.270) P=0.115	-0.452 (-1.174, 0.270) P=0.115	-0.087 (-1.479, 1.305) P=0.813	0.491 (-2.552, 3.534) P=0.559	-0.706 (-4.819, 3.407) P=0.274
Author/co-author problems	14	0.126 (-0.270, 0.521) P=0.502	0.376 (0.100, 0.652) **P=0.012 (3)**	-0.018 (-0.303, 0.267) P=0.893	-0.867 (-1.573, -0.161) **P=0.020(4)**	-0.218 (-0.686, 0.251) P=0.325

Notes to Appendix 4:

1. The Freeman-Tukey transformed proportions from primary studies were used in random-effects meta-regression (using STATA 'metareg' command)
2. Further details for the four significant findings. (1) A higher proportion of journal rejection was associated with a lower proportion of non-submission; proportion difference corresponding to a 10% difference in non-submission: 0.7% (0.3%, 1.1%); P<0.001. (2) Not important or negative result was more frequently used as a reason for non-publication of studies identified from protocols or other sources, compared with unpublished abstracts; proportion difference: 2.1% (0.0%, 8.1%); P=0.05. (3) Author or co-author problem was more frequently used as a reason for non-publication of trials, compared with non-publication of studies with mixed or other design; proportion difference: 3.5% (0.3%, 10.3%); P=0.012. (4) A higher proportion of author/co-author problem was associated with a lower response rate; proportion difference corresponding to a 10% difference in author/co-author problem: 0.2% (0.01%, 0.6%); P=0.02.
